# Synergistic Sustained Drug-Release System Based on Immobilized *Rhamnus frangula* L. Phytoextract into Layered Double Hydroxide Covered by Biocompatible Hydrogel

**DOI:** 10.3390/pharmaceutics15071888

**Published:** 2023-07-05

**Authors:** Ana-Lorena Neagu, Anamaria Zaharia, Octavian Dumitru Pavel, Alina Tîrşoaga, Iulia Elena Neblea, Sorin Viorel Dolana, Carmen Elena Ţebrencu, Tanta-Verona Iordache, Andrei Sârbu, Rodica Zăvoianu

**Affiliations:** 1 National Institute for Research and Development & Chemistry and Petrochemistry—ICECHIM, Bucharest, Spl. Independentei No. 202, Sector 6, 060021 Bucharest, Romania; ana-lorena.ciurlica@icechim.ro (A.-L.N.); anamaria.zaharia@icechim.ro (A.Z.); iulia.neblea@icechim.ro (I.E.N.); sorin.dolana@icechim.ro (S.V.D.); tanta-verona.iordache@icechim.ro (T.-V.I.); 2 Department of Bioresources and Polymer Science, Faculty of Chemical Engineering and Biotechnology, University Politehnica of Bucharest, Str. Gh. Polizu No. 1–7, Sector1, 011061 Bucharest, Romania; 3 Faculty of Chemistry, Research Center for Catalysts and Catalytic Processes, University of Bucharest, Bd. Regina Elisabeta No. 4-12, S3, 030018 Bucharest, Romania; octavian.pavel@chimie.unibuc.ro (O.D.P.); alinat@gw-chimie.math.unibuc.ro (A.T.); 4 Commercial Society for Medicinal Plant Research and Processing Plantavorelsa, Str. CuzaVoda, 46, Jud. Neamt, 610019 Piatra Neamt, Romania; carmen@plantavorel.ro; 5 Department of Chemical Sciences, Romanian Academy of Scientists, Str. Ilfov No. 3, S5, 050045 Bucharest, Romania

**Keywords:** layered double hydroxide, poly (ethylene glycol) diacrylate, composite hydrogel, phytoextract, slow release

## Abstract

This work focuses on the synergetic effect obtained by immobilization of *Rhamnus frangula* L. *(RfL)* phytoextract in layered double hydroxides (LDHs) matrixes and their subsequent encapsulation into biocompatible hydrogels (HG). In this respect, the LDHs were used as hosts for the immobilization of the phytoextract by a reconstruction method, after which the LDHs*RfL* were embedded into biocompatible hydrogel (HG) matrixes, based on polyethylene glycol diacrylate (PEGDA), by a radical polymerization reaction. The resulted biocompatible hydrogel composites were characterized by modern methods, while the swelling and rheology measurements revealed that the HG composites steadily improved as the content of *RfL* phytoextract immobilized on LDHs (LDHs*RfL*) increased. The following in vitro sustained release of the *RfL* phytoextract was highlighted by measurements at pH 6.8, in which case the composite HGs with LDHs*RfL* presented an improved release behavior over the LDHs*RfL*, thus, underlining the synergistic effect of PEGDA network and LDH particles on the slow-release behavior. The kinetic models used in the *RfL* release from composite HGs clearly indicate that the release is diffusion controlled in all the cases. The final composite HGs described here may find applications in the pharmaceutical field as devices for the controlled release of drugs.

## 1. Introduction

The classical drug-delivery systems suffer from some drawbacks, among which the most important is the incapacity to provide a long-sustained drug effect in time [1,2,3]. In order to solve this big drawback and to release the drug in a controlled manner so it is provided efficiently to target affected sites in the human body, many systems were developed based on inorganic nanoparticles [4], carbon nanostructures [5], liposomes [6], polymeric nanoparticles [7], etc. Among these systems, very convenient ones were proved to be layered double hydroxides (LDH), in which the drug is included in the interlayer space, and so its diffusion is slowed [3].

Layered double hydroxides (LDH) are two-dimensional (2D) materials, belonging to the class of hydrotalcite (HT) or anionic clays. LDHs are formed by a bivalent metal ion, M^2+^ (such as Mg^2+^, Ca^2+^, Zn^2+^, and Ni^2+^), coordinated to six hydroxyl groups, for which part the bivalent cations are substituted by trivalent cations, M^3+^ (such as Al^3+^, Fe^3+^, Cr^3+^, and In^3+^). Such an ion-exchange process results in the formation of positively charged layers, whose net charge is compensated, to maintain global electroneutrality, by the presence of anions that can participate in ion-exchange processes (A^n−^, which can be functional groups such as nitrate, chloride, and sulfate), located between the layers, together with water molecules. Thus, the general formula of a layered double hydroxide can be written in a simplified way as being [M^2+^_1−x_M^3+^_x_(OH)_2_]^x+^[A_x/n_]^n−^·mH_2_O [8,9]. The LDH compounds possess high loading capacity, biocompatibility, stability, ion-exchanging properties, and a high surface-to-volume ratio which allows for the accommodation of small and large molecules, and, therefore, they found important applications in the modern pharmaceutical field, especially as drug carriers [3,9].

A hydrogel can be defined as a three-dimensional polymer network consisting of a main polymer chain and a hydrophilic functional group. In other words, a hydrogel is a polymeric material presenting the capability to swell and retain a very significant fraction of water within its network, but which is not soluble in water [10]. The stabilization of the hydrogel structure is the result of a cross-linking process, achieved either chemically (usually by covalent bonds) or following physical cross-linking processes [11]. Hydrogels can be formed from natural, synthetic, or semi-synthetic polymers [12,13]. Hydrogel materials are used for sustained drug delivery because they exhibit very good biocompatibility [14], drug protection [15], physicochemical tailor ability [16], and tunable control of the drug release [15]. It is important to mention that hydrogels are stimuli responsive to changes for, example, in pH medium, thanks to the presence of charged hydrophilic groups [17]. Due to all these properties, hydrogels allow the encapsulation of drugs and their slow delivery to the target [18].

The interlayer space of LDH is not closed at the edges, so that the diffusion of the encapsulated drug is rather easy. To slow down the diffusion of drugs, a solution could be the sealing of the edges with another material. Such sealing was proved to be effective in the case of zeolites when a biocompatible hydrogel was used as a sealing material [19]. Usually, the LDH and hydrogel are used for the encapsulation and slow release of synthetic drugs, such as captopril [20], ibuprofen, diclofenac, and indomethacin [21], 5-fluorouracil [19,22], cephalexin [23], etc. Much more seldom, literature and patent data describe the encapsulation of phytoextracts [24]. Therefore, it would be very interesting to produce dietary supplements, knowing that phytoextracts possess very important bioactive properties and usually less toxicity than the synthetic drugs. For instance, the bark of *Rhamnus frangula* L. contains several anthraquinones derivatives occurring both in free state and as glycosides that could be easily hydrolyzed, which are known to have therapeutic activity as a laxative that increases peristalsis in the large intestine [25].The aim of the present article was to study the encapsulation of a phytoextract of *Rhamnus frangula* L. in homemade LDHs covered with biocompatible hydrogel, based on polyethylene glycol diacrylate, to make a complex characterization of the new composites and to observe the control release of the phytoextract, in vitro, at pH 6.8, prerequisites being created for an application as dietary supplements in which the sustained bioactive effect of phytoextract is synergistically combined with the mineralization effect of LDH cations (Mg^2+^, Ca^2+^, and Fe^3+^).

## 2. Materials and Methods

### 2.1. Materials

Poly(ethylene glycol) diacrylate (PEGDA) with a molecular weight of around 700 g/mol, was purchased from Sigma-Aldrich (St. Louis, MO, USA). Tetramethylethylenediamine (TMEDA) (purity 99%) was supplied by Merck Co. (Darmstadt, Germany) and ammonium persulfate (APS) (purity 98%) was purchased from Peking Chemical Works (Beijing, China). *Rhamnus frangula* L. phytoextract was provided by our collaborator from Plantavorel (Piatra Neamţ, Romania) and its characteristics are presented in the Supplementary Material. The parameters for the anthrones and derivatives (analytical reference substances) and Frangula cortex-rich extract applied in the quantification experiments are presented in Table S1 and the phytochemical characterization of the Buckthorn bark and of the phytoextract obtained by microwave-assisted extraction is presented in the Table S2. Mg(NO_3_)_2_·6H_2_O, Ca(NO_3_)_2_·4H_2_O, Fe(NO_3_)_3_·9H_2_O, Al(NO_3_)_3_·9H_2_O, NaOH, and Na_2_CO_3_ p.a. grade from Merck (Darmstadt, Germany) and distilled water were used for the preparation of LDH-type compounds. Pristine layered double hydroxide (LDHs) and *Rhamnus frangula* L. (*RfL*) phytocomplexes incorporated into layered double hydroxide (LDHs*RfL*), with the characteristics presented in Table S3, were prepared [26,27,28,29,30,31,32,33,34,35], and provided by our collaborators from the University of Bucharest. All chemicals and solvents were used without further purification.

### 2.2. Synthesis of the LDH Matrix

The LDH synthesis parameters were adapted based on own previous researches [36]. Three LDH compounds with M^2+^/M^3+^ molar ratio equal to 2/1 having different compositions of the brucite-type layer (Mg_2_Fe, Mg_2_Fe_1.8_Al_0.2_, and Mg_1.8_Ca_0.2_Fe_1.8_Al_0.2_, respectively) and CO_3_^2−^ compensation anions have been prepared using the co-precipitation method at constant pH 10 and high supersaturation. To this aim, the adequate amounts of metal nitrates were dissolved in distilled water up to a concentration of cations equal to 1.5 M, yielding solution A. A basic solution (solution B) was prepared using NaOH and Na_2_CO_3_ in order to achieve 1.33 M concentration of CO_3_^2−^ and 1.75 M concentration of NaOH. Solution A and solution B were added simultaneously, under vigorous stirring (500 rpm), in the precipitation reactor which already contained 100 mL distilled water which allowed the immersion of the pH sensor, in order to be able to maintain the pH value at 10 by appropriate adjustment of the feeding rate of the two solutions. After finishing the addition of the nitrate solution, a vertical condenser was mounted on top of the reactor and the gel was aged at 60 °C for 18 h. After aging, the solid was separated by vacuum filtration and washed with distilled water (to remove the NaNO_3_ formed as byproduct during the co-precipitation) until the conductivity of the washing water fell below 100 μS/cm. The recovered solid was dried at 90 °C for 24 h. Depending on the composition of the brucite-type layer: Mg_2_Fe, Mg_2_Fe_1.8_Al_0.2_, and Mg_1.8_Ca_0.2_Fe_1.8_Al_0.2_, respectively, the resulting LDH samples were designated as H1, H2, and H3.

### 2.3. The Immobilization of RfL Phytocomplex in the LDH Matrix

After drying, the samples H1, H2, and H3 were calcined under air flow at 450 °C, for 18 h, in order to obtain the mixed oxides (C1, C2, and C3) that were used for the immobilization of *RfL* phytoextract and the reconstruction of the LDH structure. The reconstruction was performed by immersing the mixed oxides in the extractive solution of *RfL* at a liquid/solid ratio equal to 6/1, over1 h, followed by separation of the resulting solid by filtration and washing with an amount of distilled water equal to the amount of liquid extract used in the reconstruction. Afterwards, the solids designated as R1, R2, and R3 were dried under vacuum at 40 °C for 24 h, and supplied for being incorporated in hydrogel.

### 2.4. Synthesis of Nanocomposite Hydrogels Based on PEGDA and LDHs with Immobilized RfL Phytoextract

**Figure 1:**
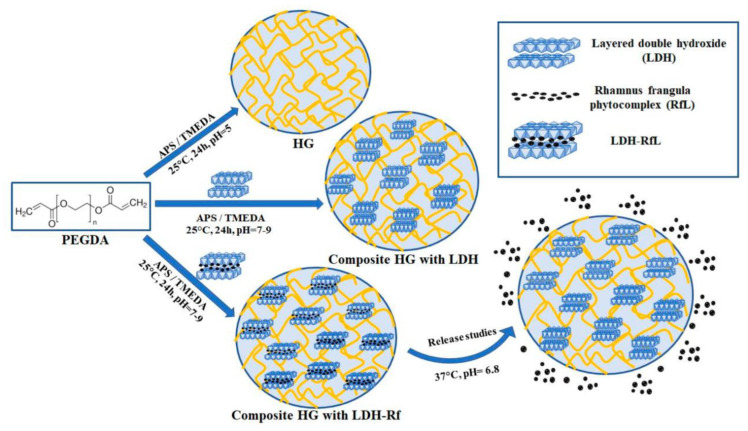
Synthesis process of the PEGDA composite hydrogels containing *RfL* phytoextract immobilized in the layered double hydroxide matrix.

The composite hydrogels (HGs) synthesis, based on polyethylene glycol diacrylate (PEGDA) and three different types of layered double hydroxide (LDHs) or *RfL* Phytoextract immobilized in LDHs matrix (LDHs*RfL*), was performed by in situ radical polymerization, in aqueous media, initiated with ammonium persulfate (APS) and tetramethylethylenediamine (TMEDA) (Figure 1). For this, the calculated quantities of pristine LDHs/LDHs*RfL* (10, 20, and 30 wt % based on PEGDA) were ultrasonicated in 3 mL distilled water for 30 min. Meanwhile, the aqueous solution corresponding to the calculated quantities of APS oxidant (2 wt % APS based on PEGDA) in distilled water was prepared. Separately, the calculated amounts of PEGDA macromer (10 wt % PEGDA in 5 mL DW) together with the TMEDA catalyst (1 wt % TMEDA based on PEGDA) and 2 mL of distilled water were introduced into glass vials with a diameter of 10 mm. At the end of the ultrasonication time, the LDHs/LDHs*RfL* suspension was added over the PEGDA and TMEDA reaction mixture and mixed under magnetic stirring for 2 min at 400 rpm and room temperature. The pH of the reaction mixtures was increased from pH = 5 to pH = 7–9, after loading with LDHs/LDHs*RfL* dispersions. Afterward, the aqueous solution of APS oxidant was also added and the reaction mixture was further homogenized by magnetic stirring for 2 min at 400 rpm and under an inert atmosphere of N_2_ gas, at room temperature. The vials were sealed with rubber seals and placed at 37 °C, for 24 h. At the end of the reaction, the vials were broken and the obtained composites hydrogels were cut into small disks of approximately 5 mm thickness. For the purification step, these disks were immersed into an excess of distilled water (DW) for seven days at room temperature; the water was changed daily in order to remove the unreacted products. After this purification step, the swollen composite HGs were dried at 40 °C, until they reached a constant weight, obtaining the xerogel [22,37,38]. Furthermore, a simple hydrogel based on PEGDA was prepared as a reference; in this case, the LDSs/LDHs*RfL* was not added in the polymerization reaction mixture.

The corresponding amounts of PEGDA, APS, TMEDA, and LDSs/LDHs*RfL* employed in each experiment and the sample codes for the final HGs composites are presented in Table 1.

### 2.5. Equilibrium Swelling Degree (ESD) Determination

The purified and swollen HG composite-disk from each sample was kept in DW for seven days at 24 °C, the water being changed daily in order to remove the unreacted compounds. After this period, the HG composite was removed from the liquid and the excess of water was wiped with filter paper, and weighed (*W_hydrogel_*). It was then dried in an oven (LabTech Vacuum Drying Oven LVO-2030) at 40 °C until it reached a constant weight, and after that the weight of the HG–xerogel disc (*W_xerpgel_*) was determined. The equilibrium swelling degree (*ESD*) was calculated as the ratio between the amount of water absorbed by the hydrogel during the purification and swelling period and the amount of dry polymer, according to Equation (1).

(1) $$ESD=\left[\frac{\left(w_{hydrogel}-w_{xerogel}\right)}{\left(w_{xerogel}-w_{LDH}\right)}\right]*100,[\frac{g\;water}{g\;xerogel}]$$

where *W_LDH_* is the mass of *LDHs* and was calculated according to Equation (2):

(2) $$w_{LDH}=\%LDH\times w_{xerogel}/100,[g]$$

where %*LDH* is the percent of *LDH* amount added to the sample, calculated to the amount of PEGDA.

The analysis of the release pattern was performed by fitting the obtained data from the in vitro *RfL* release into four mathematical models for drug release [1,2,3] as follows:

(I) Zero-order model:

(3) $$F=k_{0}\cdot t$$

(II) First-order model:

(4) $$F=100\cdot (1-e^{-k_{1}\cdot t})$$

(III) Simple Higuchi model:

(5) $$F=k_{H}\cdot t^{0.5}$$

(IV) Linear logarithm form of Korsmeyer–Peppas model:

(6) $$\log\frac{M_{t}}{M_{\infty }}=\log\left(k\right)+n\cdot log(t)$$

where *F* is the amount of drug released in time *t*, *k*_0_ is the zero-order release constant in units of concentration/time, *k*_1_ is the first-order release constant, *k_H_* is the Higuchi dissolution constant, *M_t_*/*M*_∞_ is the fraction of drug released at time *t*, *k* is the kinetic constant characteristic of the drug-polymer system, and *n* is the release coefficient that indicates the type of diffusion mechanism.

### 2.6. Characterization Methods and Instruments

The structure of the synthesized composite hydrogels was studied by attenuated total reflectance Fourier transformed infrared (ATR-FTIR) spectroscopy, diffuse reflectance UV-Vis (DR-UV-Vis), thermogravimetric analysis/differential thermal gravimetric (TGA/DTG) measurements, X-ray diffraction (XRD), and scanning electron microscopy (SEM).

Identification of functional groups: The attenuated total reflectance Fourier transformed infrared (ATR-FTIR) spectra were recorded with Jasco FT/IR-4700 spectrometer (Jasco, Tokyo, Japan) equipped with diamond crystal. The scanning speed in the spectral domain 4000–400 cm^−1^ was 128 scans/min, at a resolution of 4 cm^−1^. The triangle apodization was used for peak fitting.

Identification of structural interactions: Diffuse reflectance UV-Vis (DR-UV-Vis) spectra were recorded in the range 825–225 nm at 0.5 nm data interval and a scan speed of 1000 nm/min using Jasco V-650 spectrometer (Tokyo, Japan) equipped with an integration sphere and spectral on as white reference.

Thermal behaviour of the samples was investigated using a digitally controlled MOM thermal analysis setup (Q-1500D Derivatograph). An amount of 50–70 mg dried sample was placed into an open alumina crucible and heated under static air atmosphere from room temperature to 1000 °C with a heating rate of 5 K/min. Differential thermal analysis (DTA) signal was recorded using alumina powder as standard.

Identification of morphological changes: XRD patterns were obtained on a Shimadzu XRD 7000 diffractometer (from Shimadzu, Kyoto, Japan) using Cu Kα radiation (λ = 1.5418 Å, 40 kV, 40 mA) at a scanning speed of 0.02° min^−1^ in the 5–75° 2θ range.

The sample appearance: SEM images of the previously swollen and freeze-dried (2.5 Free Zone Labconco freeze dryer) composite hydrogel samples were recorded on the fracture surface using TM4000 plus II, table-top instrument and an acceleration voltage of 10 kV (image signal: BSE). Sample preparation consisted of immobilizing the material on a double-sided carbon tape, and coating with gold (~5 nm, with sputter coater Q150R ES Plus—Quorum) to increase the conductivity.

The rheological measurements were performed on HR 20 Discovery Hybrid Rheometer (TA Instruments, New Castle, DE, USA), using a 40 mm parallel plate geometry. The viscoelastic parameters, such as storage (G′) and loss (G″) moduli, were measured as a function of angular frequency range 0.1–600 rad/s at 1% strain and a constant temperature of 25 °C. Two measurements were taken for each composite hydrogel sample, and the average values and the error were reported.

Slow-release assessment consisted of the evaluation of the amount of *RfL* phytoextract released from the LDHs and PEGDA–LDHs composite HGs. For this, 0.2 mg of sample were dispersed into 10 mL of media at a pH = 6.8 phosphate buffer solution (prepared by mixing 3 volumes of 0.1 M HCl with 1 volume of a 0.20 M solution of Na_3_PO_4_·12H_2_O and adjusting, with 0.1 M HCl to a pH of 6.8 ± 0.05) whereas the controlled release of *RfL*phytoextract was monitored using UV–VIS. The UV–Vis analysis for slow-release assessments was performed on Thermo Scientific™ Evolution™ 260 Bio UV–Vis spectrometer (Walthman, MA, USA), by measuring the absorbance of the solution at λ = 340 nm (compounds from the *RfL* phytoextract absorb at 340 nm). The values were recorded at a temperature of 37 °C in the range of 200–800 nm using 10 mm quartz cuvettes filled with 2 mL of solution. The released amount of *RfL* phytoextract was determined from the corresponding calibration curve obtained using standard *RfL* phytoextract solution with known concentrations (Figure S2). The measurements were conducted in duplicate for reproducibility reasons and used to determine *RfL* phytocomplex-release profile.

The metal content in the solid samples H1, H2, H3, R1, R2, and R3 was determined by inductively coupled plasma atomic emission spectroscopy (ICP-AES), using a Liberty 110 spectrometer from Varian (Palo Alto, CA, USA).

## 3. Results and Discussion

### 3.1. Identification of the Interactions between the PEGDA Matrix and LDH-RfL by FT-IR Spectroscopy

**Figure 2:**
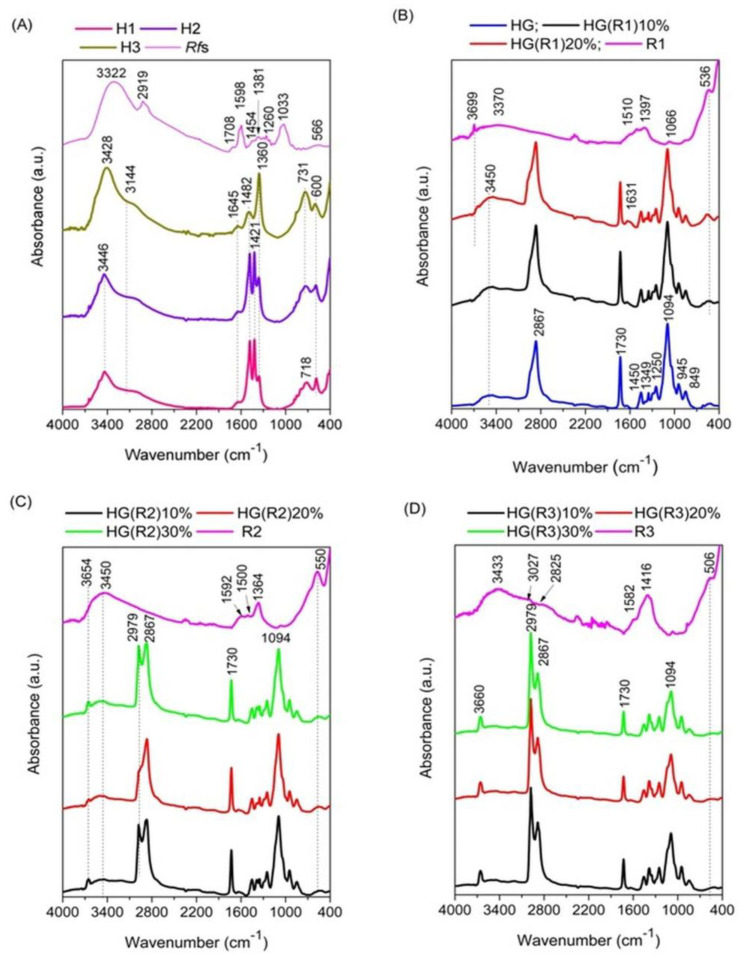
ATR-FTIR spectra of the as-prepared (before calcinations) pristine LDHs: H1, H2, and H3 and the dried phytocomplex *Rfs* (**A**), the spectra of sample R1 containing *RfL* phytocomplex immobilized by reconstruction in H1 matrix and the hydrogel-encapsulated HG(R1) samples with different concentrations of R1—10% and20% compared to the dried HG (**B**); the spectra of sample R2 containing *RfL* phytocomplex immobilized by reconstruction in H2 matrix and the hydrogel-encapsulated HG(R2) samples with different concentrations of R2—10%, 20%, and 30% (**C**); the spectra of the sample R3 containing *RfL* phytocomplex immobilized by reconstruction in H3 matrix and the hydrogel-encapsulated HG(R3) samples with different concentrations of R3 (10%, 20%, and 30%) (**D**).

All ATR-FTIR spectra of the pristine LDH samples, H1-H3 (Figure 2A) show the characteristic absorption maximum for stretching vibrations in OH groups at 3446–3428 cm^−1^ accompanied by a shoulder at 3144 cm^−1^ due to the hydrogen bonds created with the carbonate anions located in the interlayer region [36], along with the band for water deformation in the interlayer at 1645 cm^−1^ and a band characteristic to Fe-O bond stretching vibrations at 600 cm^−1^ [39]. In the region 1600–1300 cm^−1^, there are some bands related to the vibrations in the carbonate compensation anions. The spectra of samples without calcium in the brucite-type layer (H1 and H2) show two intense absorption bands at 1482 and 1421 cm^−1^ and a lower intensity band at 1360 cm^−1^, while in the spectrum of H3, the most intense band is the bandat 1360 cm^−1^ which is characteristic of bidentate configuration of the carbonate anion [40], the band at 1482 cm^−1^ has a lower intensity and the band at 1421 cm^−1^ is missing. The band appearing in the region 800–600 cm^−1^ related to M-O-H (M = metal) deformation vibrations [41,42,43] is located differently, depending on the cationic composition of the samples; thus, for the sample containing only Mg(II) and Fe(III) (e.g., H1) the maximum appears at 718 cm^−1^ while for the other samples the maximum is shifted to higher wavenumbers (731 cm^−1^). The spectrum of the dried phytoextract Rfs (Figure 2A) shows a broad intense band at 3322 cm^−1^ (stretching -OH) accompanied by a shoulder at 2919 cm^−1^ (stretching of aromatic CH), and typical bands for substituted quinones in the fingerprint region [44,45].

In the spectra of the samples R1–R3 obtained after the immobilization of the *RfL* phytoextract by reconstruction in the LDH matrices (LDHs*RfL*) (Figure 2B–D), the bands characteristic to the pristine LDH were significantly altered and the bands of *RfL* cannot be neatly separated from those of the corresponding matrix. However, it may be seen that the position of the maxima is different depending on the cationic composition of the LDH matrix, suggesting that there are specific interactions between the organic phase from the phytoextract and the cations in the brucite-type layer. For all three samples, the most intense band is located in the region characteristic to M-O vibrations below 600 cm^−1^, suggesting that the reconstruction was incomplete and there are still oxide phases present in the solids. This fact was also indicated by the results of the XRD analysis. The bands in the region 1600–1300 cm^−1^ where there is an overlapping of the bands characteristic to LDH structures and those characteristic to the phytocomplex have significantly lowerintensity compared to the same region for H1–H3 spectra.The characteristic maxima for the spectrum of the dried hydrogel HG are displayed in Figure 2B. The FTIR spectra of simple hydrogel presented the characteristic peaks of polyethylene glycol diacrylate macromer: stretching vibrations of –CH_2_– group at around 2880 cm^−1^ and of C–O–C groupataround 1100 cm^−1^. Also, the FTIR spectra of HG displayed the characteristic peaks of PEGDA: at 1248 cm^−1^, characteristic to the O–H stretching vibrations, the peaks around 1350 and 1450 cm^−1^ characteristic to the C–H wagging and bending of –O-Hand those around 1730 cm^−1^ and around 1630 cm^−1^, which are characteristic to the carbonyl group –C=O and to the asymmetric stretching of the −C=C−, from the acrylate group of PEGDA [41]. The encapsulation of R1, R2, and R3 (Figure 2B–D) in the hydrogel maintains the specific peaks of HG, but their intensity varies depending on the amount of LDHs*RfL* solid incorporated. The cation composition of the brucite-type layer affects differently the reticulation of the hydrogel. After the encapsulation of R1, whose matrix contains only Mg and Fe, there is no splitting of the band at2867 cm^−1^ of the hydrogel. The encapsulation of R2 (which contains Mg, Fe, and Al) leads to the occuring of two new bands, one intense at 2979 cm^−1^ and a weak one at 3654 cm^−1^, while for the encapsulation of R3 (which contains Mg, Ca, Fe, and Al) the new band at 2979 cm^−1^ is more intense than the bandat 2867 cm^−1^ and the band that was noticed at 3654 cm^−1^ for R2 containing composite is shifted to 3660 cm^−1^ and its intensity is higher. The FTIR spectra support the idea that no new covalent bonds appear during the immobilization of phytoextract in LDHs and, subsequently, in hydrogel.

**Figure 3:**
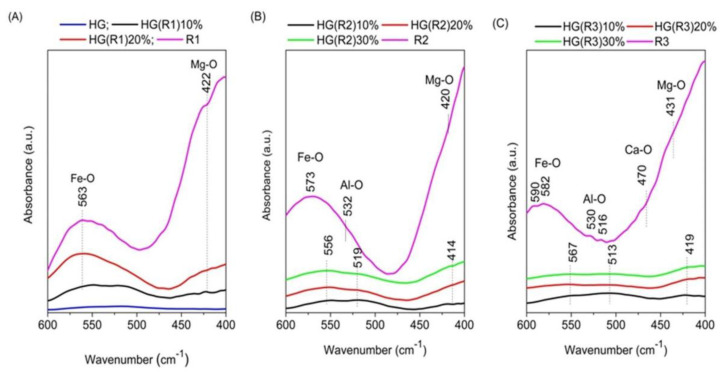
Five-fold amplified ATR-FTIR spectra of the sample R1 containing *RfL* phytocomplex immobilized by reconstruction in H1 matrix and the hydrogel-encapsulated HG(R1) samples with different concentrations of R1 (10% and 20%) compared to the dried HG (**A**); the spectra of sample R2 containing *RfL* phytocomplex immobilized by reconstruction in H2 matrix and the hydrogel-encapsulated HG(R2) samples with different concentrations of R2—10%, 20%, and 30% (**B**); the spectra of the sample R3 containing *RfL* phytocomplex immobilized by reconstruction in H3 matrix and the hydrogel-encapsulated HG(R3) samples with different concentrations of R3—10%, 20%, and 30% (**C**).

In order to highlight the modifications of the spectra in the region 600–400 cm^−1^, where the bands characteristic to M-O vibrations appear, the spectra of hydrogel-encapsulated samples containing different amounts of R1, R2, and R3 were five-fold amplified in that region and represented separately in Figure 3.

As it may be seen in Figure 3A, the neat hydrogel does no present absorption bands in the investigated domain. For all the hydrogel-encapsulated samples the bands are very weak due to the low amount of incorporated LDH-*RfL.* For the sample R1 which contained only Mg and Fe in the LDH matrix, the positions of the characteristic absorption maxima after its encapsulation in the hydrogel were shifted to lower wavenumbers only at the lowest concentration of R1, suggesting that the new bonds created between R1 and the polymer were very weak. The incorporation of the samples R2 and R3 into the hydrogel leads to the modification of the positions of the maxima characteristic to the neat LDH-*RfL* samples (Figure 3A,B), suggesting that Al-O and Fe-O bonds are formed with the polyether groups of the polymer.

### 3.2. Identification of the Interactions between the PEGDA Matrix and LDH-RfL by DR-UV-Vis Spectroscopy

**Figure 4:**
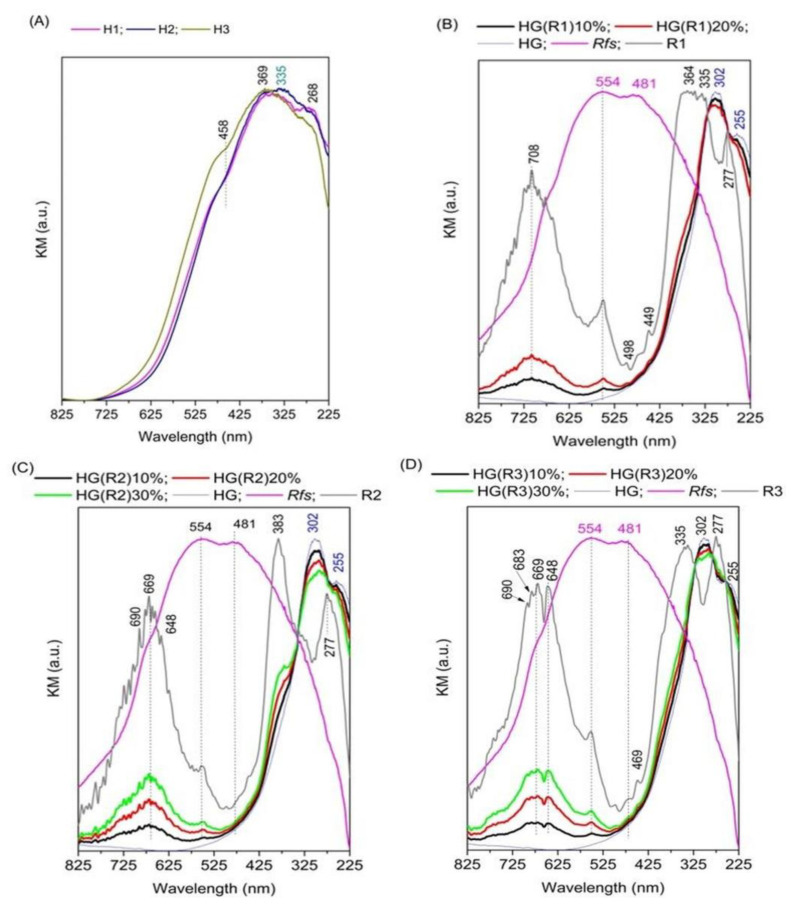
DR-UV-Vis spectra of the as-prepared (before calcinations) pristine LDHs: H1, H2, and H3 (**A**), the spectra of sample R1 containing *RfL* phytoextract immobilized by reconstruction in H1 matrix and the hydrogel-encapsulated HG(R1) samples with different concentrations of R1—10% and 20% compared to the dried *RfL* phytoextract (Rfs) and dried HG (**B**); the spectra of sample R2 containing *RfL* phytoextract immobilized by reconstruction in H2 matrix and the hydrogel-encapsulated HG(R2) samples with different concentrations of R2—10%, 20%, and 30% compared to*RfL* and dried HG (**C**); the spectra of the sample R3 containing *RfL* phytoextract immobilized by reconstruction in H3 matrix and the hydrogel-encapsulated HG(R3) samples with different concentrations of R3—10%, 20%, and 30% compared to Rfs and dried HG (**D**).

The DR-UV-vis spectra of the pristine LDHs (Figure 4A) present the most intense absorption maximum at 335–369 nm characteristic to Fe in octahedral symmetry [3,46,47,48] and the maximum at 268 nm corresponding to O→Fe^3+^ charge-transfer characteristic for α-Fe-O(OH) [47].

The DR-UV-Vis spectrum of the dried *RfL* phytoextract (Rfs) shows a broad absorption band with two maxima at 554 and 481 nm (Figure 4B–D) due to the electronic transitions characteristic to conjugated aromatic systems which exist in its composition [49]. After the incorporation of *RfL* phytoextract in the LDH matrix, the charge-transfer bands characteristic to the pristine LDHs become sharper and more intense and the position of the maximum at 268 nm (Figure 4A) shifts to higher wavelength 277 nm (Figure 4B–D). The bands characteristic to the dried *RfL* phytoextract appear for all reconstructed samples and the band at 554 nm displays the highest intensity for R3 (Figure 4D, while the band at 481 nm has the same intensity for all samples. The most significant changes of the DR-UV-Vis spectra compared to those of the parent LDHs are noticed in the region characteristic to d-d and n-π transitions, where new bands appear in the region 725–600 nm due to the complexation of the organic moieties from the *RfL* phytoextract to the Fe sites from the LDH matrix. The positions and intensities of the maxima differ slightly depending on the composition of the LDH matrix. Thus, for the sample R1 whose matrix contains only Mg and Fe, there is one sharp maximum at 708 nm (Figure 4B), for the sample R2 whose matrix contains Mg, Fe, and Al there are two intense maxima at 690 and 669 nm and two resembling a shoulder at 683 and 648 nm (Figure 4C), while for the sample R3 whose matrix contains Mg, Ca, Fe, and Al there are two well-defined intense maxima at 669 and 648 nm and two maxima resembling a shoulder at 690 and 683 nm (Figure 4D). This fact indicates that the components of the R*fL* phytoextract have different interactions in the ligand field of the solid depending on the chemical composition of the matrix. Since stronger interactions are characterized by lower wavelengths of the absorption maximum it may be inferred that the *RfL* phytoextract is stronger held in the matrix of R3. After the incorporation of R1, R2, and R3 into the hydrogel, the spectra of the resulting solids display the characteristic bands of R1, R2, and R3 with a lower intensity depending on their concentration in the hydrogel composite. For the samples prepared with R1 the absorption band at 335–369 nm characteristic to Fe in octahedral symmetry is noticed as a shoulder at 348 nm in the spectra of HG(R1)10% and 20%, respectively, while the maximum at 277 nm is overlapped by the band characteristic to the hydrogel (Figure 4B). A similar effect was noticed for R2 samples encapsulated in composite HG, where the shoulder (corresponding to the maximum at 383 of the neat R2 is noticed at 370 nm (Figure 4C). For the samples prepared with R3, whose LDH-matrix contains Mg, Ca, Fe, and Al, this shifting was not noticed, suggesting a better stabilization of the solid in the hydrogel structure.

### 3.3. Thermogravimetric (TGA/DTG) Investigation of the PEGDA/LDH-RfL Composite HGs

Thermal-analysis diagrams obtained for the synthesized samples indicate specific characteristics according to the nature of each sample category: pristine and reconstructed LDHs present three main decomposition steps whereas only two are noticed for calcined LDHs (Table 2). Furthermore, the presence of hydrogel adds more complexity to the thermal behavior, namely, an overall of four thermal decomposition steps. The general evolution of all LDH samples starts with water removal followed by decarbonation and, finally, the collapse of the double-layer structure at higher temperature [50] with specific features according to the cationic substitution performed (Fe with Al and, subsequently, Mg with Ca). The samples containing *RfL* phytoextract present exothermal steps because of the oxidative degradation processes occurring for the included organic component. All samples embedded in the PEG-based hydrogel matrix exhibit also specific thermal degradation steps of the polymeric backbone [23,51,52] that can be significantly influenced by the high-temperature interaction with the iron-based inorganic content of the samples [53,54]. Detailed discussion of the thermal-analysis results, based on the Table S4 and Figure S2, is given in the Supplementary Material.

### 3.4. Morphological Characterization by XRD of the PEGDA/LDH-RfL Composite HGs

**Figure 5:**
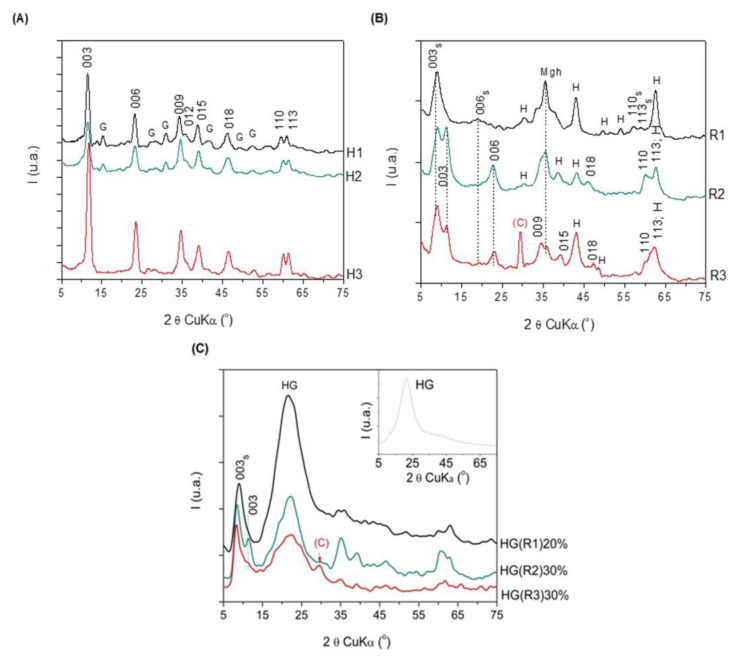
XRD patterns of the pristine LDHs (**A**), the reconstructed samples containing *RfL* phytoextract (**B**) and the hydrogel-encapsulated *RfL* phytoextract immobilized in LDH matrix (**C**); inset in (**C**)—the XRD pattern of the neat hydrogel (HG); abbreviations: (C)—calcite; G—goethite; H—hematite; Mgh—maghemite.

The XRD patterns in Figure 5A presented all the main diffraction lines characteristic to the LDH phases, the sample with the highest crystallinity (e.g., H3) being the one with quaternary composition of the brucite-type layer. In all patterns, low-intensity diffraction lines corresponding to goethite (PDF 01-081-0464) impurity (marked with G) were also noticed.

The immobilization of *RfL* phytoextract in the LDH matrix affected the crystallinity of the resulting solids as indicated by the XRD patterns in Figure 5B which show wider and lower-intensity diffraction lines compared to those noticed in Figure 5A. *RfL* incorporation led to the splitting of the diffraction lines corresponding to 003 reflection in the patterns of R2 and R3, while in the pattern of R1 this line was shifted to lower values of 2θ. For the sample R1, the diffraction lines for 006, 110, and 113 reflections were also shifted to lower values of 2θ. This fact indicated the formation of an extra layered phase having a larger interlayer space, proving the intercalation of larger-sized anions from the *RfL* phytoextract. The small value of 2θ corresponding to 003 reflection of this extra-phase denotes a degree of crystalline disorder along the c-axis, the axis on which the brucite-like layers are stacked [36]. Bands corresponding to traces of hematite (JCPDS card no. 1-087-1164) and maghemite (JCPDS card no. 39-1346) are visible in the patterns of R1–R3 samples, while the pattern of R3 shows also a diffraction line corresponding to calcite (JCPDS card no. r01-071-3699) impurity. This fact suggests that the reconstruction of the layered LDH structure was only partial, since these oxide-phase impurities were stable and did not participate in the dissolution–recrystallization process.

The diffraction patterns of the composite HGs encapsulated with *RfL* phytoextract in the LDH matrix (Figure 5C) show broad diffraction lines with low intensity. For the sample containing 80% hydrogel and 20% R1, the most intense diffraction line is the line corresponding to the hydrogel (see the inset figure), whereas for the samples HG(R2)30% and HG(R3)30%, which contain only 70% hydrogel, the most intense lines are those related to the 003s reflection of the LDHs*RfL* phase. The alteration of the intensities of the remaining diffraction lines corresponding to R1, R2, and R3 suggests that during the encapsulation in the hydrogel a dissolution–recrystallization process involving the LDHs*RfL* phase takes place.

Table 3 shows the values of the calculated lattice parameters for the LDH phases existent in all the samples. The distance between two metals cations in the brucite-like sheet is related to the value of the *α*-lattice parameter and depends only on the molar ratio between bivalent and trivalent cations M^2+^/M^3+^, while the *c*-lattice parameter depends on both this ratio and the size of the interlayer anions [36]. It may be noted that *α*-parameter increases slightly when Fe is partially substituted by Al in the samples H2 and H3. The same effect is noticed for the samples R1–R3 and the hydrogel-encapsulated samples HG-LDH*RfL.* The immobilization of the *RfL* extract in the LDH matrix leads to a decrease inα-parameter compared to the corresponding LDHs precursors, suggesting that the phytocomplex disturbs somehow the position of the cations in the brucite-type layer. The decrease in*α* is even more significant after the encapsulation of the LDH*RfL* samples in the hydrogel, indicating that the cations became involved in establishing links with the polymer. The *χ*-parameter is almost similar for the samples H1 and H2 but is significantly lower for the sample H3 which also contains Ca cations. This fact may be due to the larger ionic radius of Ca^2+^ compared to Mg^2+^, which may lead to a shrinking of the interlayer. All LDH*RfL* samples have larger *χ*-values than the corresponding parent LDHs, indicating that the phytocomplex has been accommodated in the interlayer region. The encapsulation of LDH*RfL* in the hydrogel results in a further increase in *χ* parameter.

### 3.5. Morphological Characterization by SEM of the PEGDA/LDH-RfL Composite HGs

**Figure 6:**
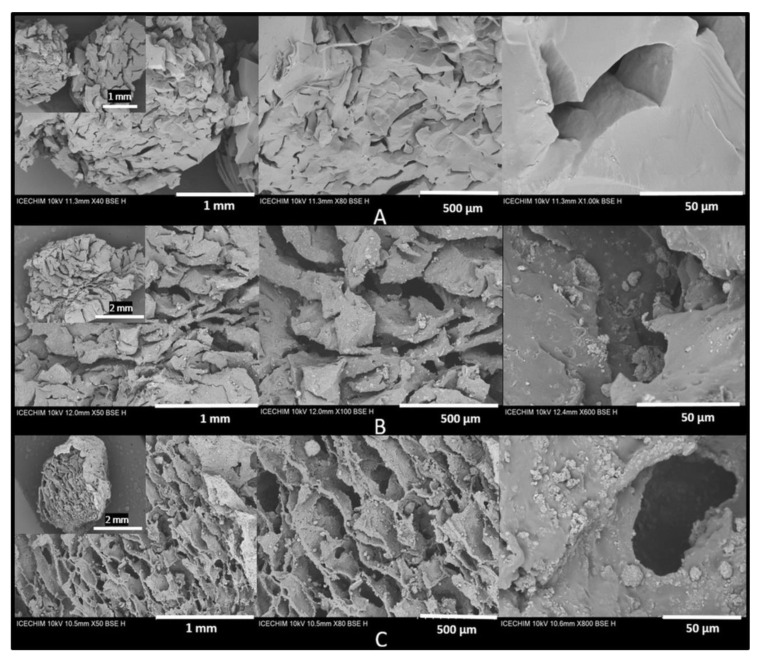
SEM micrographs of lyophilized composites HG. (**A**) control HG; (**B**) composite HG with LDHs—HG(H1)20%; (**C**) and composite HG with *RfL* Phytocomplex immobilized in the LDHs matrix- HG(R1)20%.

In Figure 6, SEM micrographs at three different magnifications of the lyophilized composite HGs with *RfL* phytoextract encapsulated in the LDHs matrix are presented. SEM micrographs were used to evaluate the morphology of all studied samples, knowing that the surface morphology has a great influence on the drug-release behavior. The micrographs clearly highlight the presence of LDHs in the structure of final HG composites. In Figure 6A, the images reveal a homogeneous morphology along with some cracks that led to the formation of porosity during the lyophilisation process of the control HG. The top surface morphology seen from above, looks like a smooth flat surface with large elongated pores. In Figure 6B, the SEM micrographs of the lyophilized HGs composite based on PEGDA and pristine LDH (HG(H1)20%), indicate the rough, homogeneous morphology and the presence of LDHs under plate-like shape agglomerates with a porosity and voids of the polymer network, together with some cracks formed during the freeze-drying process. Regarding the HG composites with *RfL* phytoextract encapsulated in the LDHs matrix (HG(R1)20%), the Figure 6C, reveals also a rough, homogeneous surface and a macro porous morphology with open and interconnected channels in the range of tens of microns. The rough and porous surface is an advantage in the drug-delivery systems [55]. Other SEM micrographs are displayed in Figure S4.

**Figure 7:**
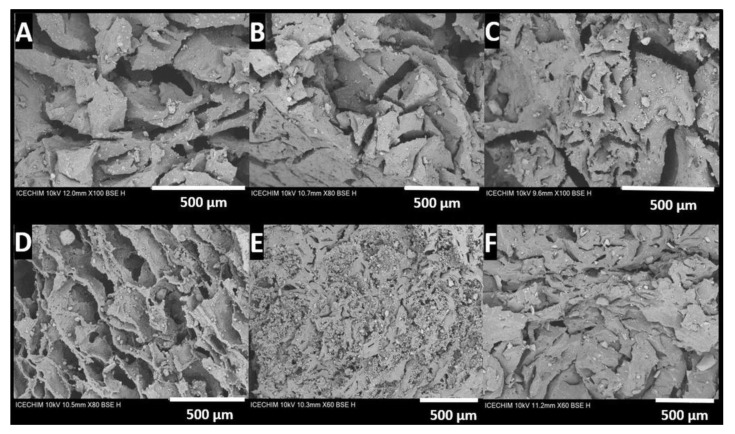
SEM micrographs of lyophilized composite HGs: (**A**–**C**) composites with LDHs without phytoextract, namely: (**A**) HG(H1)20%, (**B**) HG(H2)30%, (**C**) HG(H3)30% and HG composites with *RfL* phytoextract immobilized in the LDHs matrix, namely: (**D**) HG(R1)20%, (**E**) HG(R2)30%, and (**F**) HG(R3)30%.

The porosity and the homogeneous distribution of LDH of all studied samples were also revealed in cross-section images (Figure 7 and Figure S3 in Supplementary Material). Looking into the detailed structures in the SEM micrographs, it is obvious that the surface of HG composites with incorporated LDHs (Figure 7A–C) exhibits a less homogeneous and more agglomerated structure (Figure 7D–F), thereby suggesting that the *RfL* phytoextract immobilized in the LDHs matrixand encapsulated in the HGs leads to an improvement in the dispersion of LDHs particles inside the polymeric network Therefore, it is relevant to say that the improvement of the dispersion with the *RfL* phytoextract immobilized in the LDHs matrix inside the polymer network may somehow be due to *RfL* phytoextract anions intercalated into the interlayer space of the LDHs, which caused the surface of the LDH to be more hydrophilic, leading to an improved compatibility of LDH particles with the hydrophilic polymer matrix. Also, the *RfL* phytocomplex encapsulated in the interlayer space of LDHs could intercalate the LDH layers, which can also lead to a better compatibility between the LDH filler and the PEGDA network [56]. The SEM images of hydrogel composites containing LDH with and without phytoextract (Figure 7) show a porous structure with interconnectivity between the pores, which is very important for drug release. The porosity and the dimensions of pores are very different, depending on the type of LDH and phytoextract content, being, generally, over 40%.

### 3.6. Equilibrium Swelling Degree

**Figure 8:**
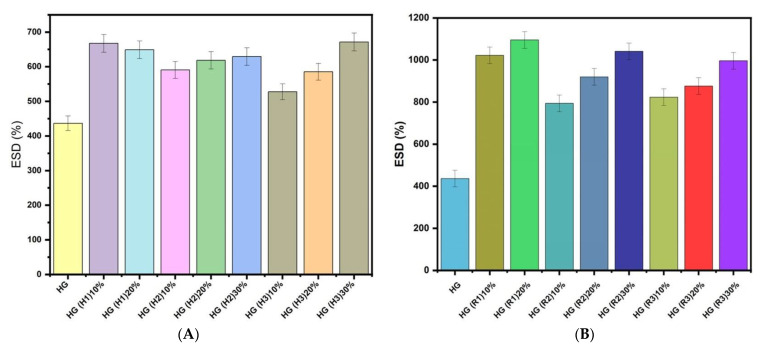
Equilibrium swelling degrees of the synthesized HG composites: (**A**) HG composites containing pristine LDH; and (**B**) HG composites containing *RfL* phytoextract immobilized in the LDH matrix.

The equilibrium swelling degree (ESD) measurements in distilled water at room temperature are presented in Figure 8 for all studied samples. The equilibrium water content of the PEGDA hydrogels increased with the presence of the LDH filler (Figure 8A). This increase was more pronounced after immobilization of *RfL* phytoextract in the LDHs matrix (Figure 8B). The increase in water absorption as a result of *RfL* phytoextract immobilization in the LDHs particles and incorporation in the hydrogel structures is attributed to the presence of the phenolic compounds that limits the chain propagation (phenols might act as a chain transfer agent) [38]. The results show that a higher LDHs content in the polymer network leads to higher ESD of the HG composites. A possible explanation of this behaviour can be the intercalation of the LDH layers which led to a good dispersion in the polymer network and the yielding of more intercalate nanolayers increases the hydration ability of the OH group. A similar result was described by Lee and Chen in the case of poly (acrylic acid-co-n-isopropyl acrylamide)/intercalated hydrotalcite nanocomposites hydrogels [57].

Also, a difference in ESD can be observed depending on the type of encapsulated LDHs. Thus, in the case of H1(Mg_2_Fe) and H1 loaded with *RfL*(R1) encapsulated in the gel, a degree of swelling above 1000% can be observed. Due to the structure, the hydrogel acquires an affinity for water molecules and improves the swelling ratios of the final composite hydrogels. In the case of the pairs of composite HGs with LDH samples, namely, HG(H2), HG(R2) that contain LDH based on Mg, Fe, and Al, and HG(H3) and HG(R3) that contain LDH based on Mg, Ca, Fe, and Al; the swelling degree is slightly reduced compared to that of the LDH samples, which contain only Mg and Fe. It is well known that the presence of ions as Mg^2+^, Ca^2+^, and Al^3+^ in the hydrogel structure leads to a decrease in the swelling degree. Shivakumara and Demappa [58] have suggested that the bivalent cations develop strong interactions with the polymeric network and are able to cause further shrinking of the hydrogel network. However, in our case, the ESD increased with the presence of pristine LDHs and reconstructed LDHs*RfL* in the gel network. Enhancing the content of LDHs and LDHs*RfL* leads to an increase in ESD, in almost all the cases, showing again that intercalated structures are formed. Only in the case of HG(H1)10% and HG(H1)20% a slight decrease was noticed, revealing that the enhancement of cations concentration also plays a role in the hydration process.

### 3.7. Rheological Behavior

**Figure 9:**
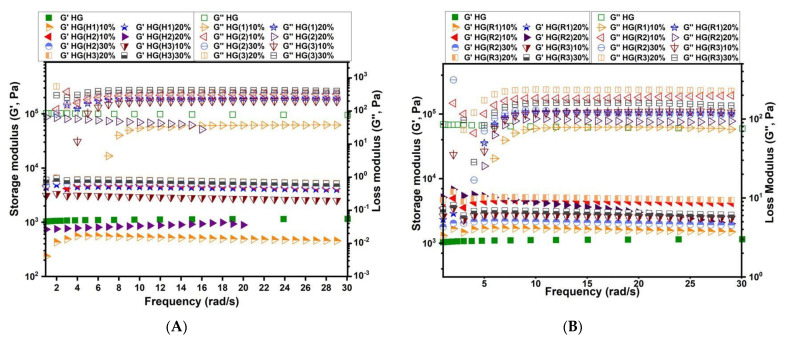
Viscoelastic properties determined through frequency sweep measurements for the composite HGs containing pristine LDH (**A**) and containing *RfL* phytoextract immobilized in the LDH matrix (**B**).

The rheological behavior of the equilibrium swollen composite HGs was assessed by measuring the influence of the angular frequency on elastic modulus (G′) and loss modulus (G″). The frequency sweep measurements revealed that the elastic modulus (G′) was greater than the loss modulus (G″) over the whole investigated range, thus, confirming the crosslinked character of the hydrogels as presented in Figure 9. The fact that G′ is higher than G″ could be correlated to low numbers of network imperfections [59]. Therefore, one can notice that the addition of LDHs particles improved the mechanical properties of the composite HGs, the increasing of G′ being in the order: control HG < composite HGs containing pristine LDH < composite HGs containing *RfL* phytoextract immobilized in the LDH matrix. The gel strength increase may be explained by a better dispersion of the LDH particles in the PEGDA network, thus, leading to enhanced interactions and, consequently, to improved mechanical properties of the final hydrogels.

As stated in the ESD measurements presented above, the interaction between the LDHs particles and the PEGDA network influences the swelling behavior of the composite HGs. Therefore, in our case, the G′ values obtained for the neat HG displayed the same tendency as ESD, the control HG with a lowest ESD (HG, Figure 8) showing the lowest G′ (Figure 9), while the composite HGs with higher ESD namely HG(R2)30%,and HG(R3)30% (see Figure 8B) showed also higher G′ values (Figure 9B). Thus, the intercalated/dispersed LDH nanolayers in the PEGDA network, rendered possible the obtaining of a final composite HG with a stronger hydration capacity (due to the OH groups from LDH structure), and, at the same time, exhibiting a better elastic modulus (Figure 9A,B) than the neat HG. The loss tangent (tan δ = G″/G′) was calculated from the G′ and G″ values at 0.955 rad/s (~1 Hz). The values of a loss tangent lower than 1 for all the composite HGs represented an additional confirmation of their crosslinked character [60]. These results are in agreement with the SEM observations and the ESD measurements, where the *RfL* phytoextract immobilized in the LDHs matrix inside the polymer network showed a rough homogeneous surface and a macroporous morphology with open and interconnected channels and a stronger water-retention capacity compared with the neat HG.

### 3.8. Controlled-Release Experiments

**Figure 10:**
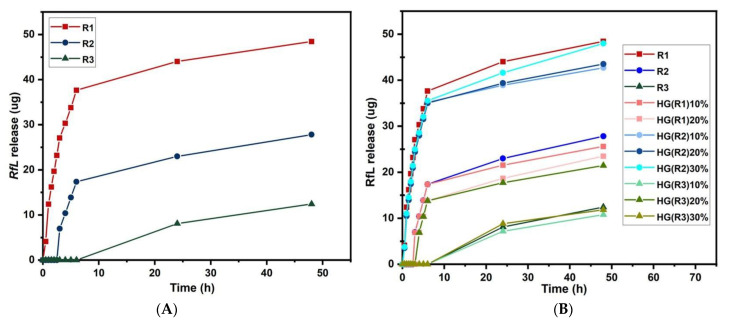
The release profile of RfL phytoextract (**A**) from composite HGs containing pristine LDH and (**B**) from composites HGs containing RfL phytoextract immobilized in the LDH matrix; over 24 h.

The results displayed in Figure 10 show the release rate of the *RfL* phytoextract at pH 6.8 for LDHs*RfL* and HGs with LDHs*RfL* samples. As one can see in the Figure 10A, the release rate of RfL phytoextract from LDHs not covered with HGs was higher for the R1 sample and smaller for the R2 and R3 samples, in the same time interval. In the case of R2 and R3, a delayed release is observed, showing the good encapsulation of phytoextract in LDH. Thus, the amount of released *RfL* depends on the composition of the LDH samples. Therefore, the *RfL* release was influenced by the interaction between immobilized *RfL* molecules and the bulk surface of LDH particles [61].

The release rate of the *RfL* phytoextract in the HG composites depending on time is shown in Figure 10B). For all the HG composites formulations, a sustained slow-release behavior was observed, with practically no burst effect. The slow-release rate in the case of the HG composite samples certificates the presence of the interactions between PEGDA chains, LDH particles, and *RfL* molecules [38]. The release rate of the *RfL* in the composite HGs decreased with the increase in the percentage of LDHs, in the case of the HG composite with encapsulated R1. The release rate obtained for the composite HG with encapsulated R1 presented an inverse tendency in relation to the water-retention capacity. This fact is due most probably to the compatibility between the *RfL* phytoextract and the HGs composite. The release rate of the *RfL* phytoextract in the case of the HG composites with encapsulated R2 and R3, showed the same tendency with the water-retention capacity. This fact is due most probably to the presence of ions, such as Al^3+^ and Ca^2+^, in the HG composite structure, that lead to the decrease in the existing attractions between the *RfL* phytoextract, the LDH particles, and the HGs composite [62,63,64].

As shown in Figure 9, from rheological behavior, the presence of the LDH-*RfL* in the hydrogel network led to a higher crosslinking degree of the network (G′ increased with the LDH-RfL concentration), making possible the reduction of the burst effect, similar to the literature data [9]. An important contribution to this release behavior can also be attributed to the hydrogen bonds formed between the -C=O group of PEGDA and the -OH functional groups of the phenolic compounds from RfL phytoextract. Additionally, the interactions between the cations from LDH and the electrons from the oxygen atom of the OH group or the aromatic ring of phenols [65,66] from the RfL phytoextract may also minimize the burst release.

Very important to notice is that many HG composites, such as HG(R1)10%, HG(R1)20%, and all HG with R3, not only do not display the burst release, but instead present a delayed release, meaning that the phytoextract release begins after a while. At the same time, the release of phytoextract from HG composites is slower than that from the LDHs. These facts clearly emphasize the synergistic effect of hydrogel and the LDH system on slow-release behavior. It can be concluded that the obtained HG composites are very promising as synergistic sustained drug-release systems due to their good control over the release of *RfL* phytoextract.

Table 4 lists the release data obtained by means of the four mathematical models described in Section 2.5. This study aims to offer a better understanding of *RfL* release mechanism from LDH particles and composite HGs. The parameters obtained were fitted to each model to determine k, R^2^, and n coefficients for each sample. For all the models studied, the Higuchi and Korsmeyer-Peppas (highest R^2^ values) models were found to be more suitable than the zero-order or first-order models. This classification clearly indicates that release is diffusion-controlled in all the cases. The kinetic description is characteristic to the low-molecular-weight compounds diffusing from porous matrices, such as composite HGs. The Korsmeyer-Peppas model considers the dissolution events and the swelling behavior that is characteristic of the proposed composite HGs. The Higuchi model does not account for any dissolution of the polymeric matrix and only allows for minor changes in the shape and size of the system. The low values for R^2^ for all mathematical models prove that, most probably, two different releasing mechanisms are involved for LDHs and for hydrogel and, so, the synergistic effect is explained.

Table 4 also gives the value of the parameter n. The calculated values for the results of drug-release exponents, for a cylindrical geometry, n, varies from 0.38 to 0.48 which implies Fickian diffusion mechanism, i.e., diffusion-controlled transport. That means that the diffusion rate in slab geometry decreases continuously with time, owing to the continuous decrease in the driving concentration gradient [67,68]. Individual graphs for zero-order, first-order, Higuchi, and Korsmeyer-Peppas models, for sample HG(R2)20%, are provided as Supplementary Data (Figure S5).

## 4. Conclusions

The study proved the noncovalent nature of the interactions between the phytoextract components, LDH and hydrogel. The study on thermal stability, performed in air, proved that the LDH and phytoextract components embedded in the hydrogel exhibit specific thermal degradation steps. The alteration of the intensities of XRD diffraction lines suggests that during the encapsulation in the hydrogel a dissolution–recrystallization process involving the LDHs*RfL* phase could take place. The surface of composite HGs with incorporated LDHs exhibits a less homogeneous and more agglomerated structure than that of samples with LDHs*RfL*, suggesting that the *RfL* phytoextract leads to an improvement in the dispersion of LDHs particles inside the polymeric network and that some intercalated structures could appear. Although it is well known that the presence of ions, such as Mg^2+^, Ca^2+^, and Al^3+^ in the hydrogel structure leads to a decrease in the swelling degree, in our case, the equilibrium swelling degree of the composite hydrogels increased in the presence of pristine LDHs and reconstructed LDHs*RfL* in the gel network. A more crosslinked structure of HG composites was formed by the addition of LDHs particles, which improved the mechanical properties. Studying the release of phytoextract from composite HGs with LDH*RfL*, no burst effect was observed and many such composites presented a delayed release, which underlined the synergistic effect of PEGDA network and LDH particles on the slow-release behavior. These findings can lead to an efficient slow-release system, at the intestine pH, for slow intestinal-transit treatment. Further studies on the interactions between the components of the systems, on the elements content (EDAX), on the stability at different temperatures and pH, on the biocompatibility, and animal studies are foreseen.

## Figures and Tables

**Table 1:** The sample code, the cationic composition, and the amounts of materials used for the synthesis of the obtained HGs composites.

Sample Code	Cationic Composition of LDHs	PEGDA (g)	TMEDA (g)	APS (g)	LDHs (g)	LDHs*RfL* (g)	DW (g)
HG	0	0.5	0.00645	0.01	-	-	4.99
HG (H1)10%	Mg, Fe	0.5	0.00645	0.01	0.05	-	4.99
HG (H1)20%	Mg, Fe	0.5	0.00645	0.01	0.1	-	4.99
HG (H2)10%	Mg, Fe, Al	0.5	0.00645	0.01	0.05	-	4.99
HG (H2)20%	Mg, Fe, Al	0.5	0.00645	0.01	0.1	-	4.99
HG (H2)30%	Mg, Fe, Al	0.5	0.00645	0.01	0.15	-	4.99
HG (H3)10%	Mg, Ca, Fe, Al	0.5	0.00645	0.01	0.05	-	4.99
HG (H3)20%	Mg, Ca, Fe, Al	0.5	0.00645	0.01	0.1	-	4.99
HG (H3)30%	Mg, Ca, Fe, Al	0.5	0.00645	0.01	0.15	-	4.99
HG (R1)10%	Mg, Fe	0.5	0.00645	0.01	-	0.05	4.99
HG (R1)20%	Mg, Fe	0.5	0.00645	0.01	-	0.1	4.99
HG (R2)10%	Mg, Fe, Al	0.5	0.00645	0.01	-	0.05	4.99
HG (R2)20%	Mg, Fe, Al	0.5	0.00645	0.01	-	0.1	4.99
HG (R2)30%	Mg, Fe, Al	0.5	0.00645	0.01	-	0.15	4.99
HG (R3)10%	Mg, Ca, Fe, Al	0.5	0.00645	0.01	-	0.05	4.99
HG (R3)20%	Mg, Ca, Fe, Al	0.5	0.00645	0.01	-	0.1	4.99
HG (R3)30%	Mg, Ca, Fe, Al	0.5	0.00645	0.01	-	0.15	4.99

**Table 2:** Thermal behavior of investigated samples (RT = room temperature).

Sample	T_i_—T_f_ (°C) Δm (%)	T_i_—T_f_ (°C) Δm (%)	T_i_—T_f_ (°C) Δm (%)	T_i_—T_f_ (°C) Δm (%)
H1	RT–209 −11.41%	209–500 −22.42%	500–569 −5.01%	-
H2	RT–223 −13.20%	223–494 −22.71%	494–543 −2.79%	-
H3	RT–231 −14.00%	223–550 −19.74%	664–773 −2.92%	-
C1	RT–500 −2.4%	500–784 −1.25%	-	-
C2	RT–500 −3.2%	500–784 −1.65%	-	-
C3	RT–500 −2.5%	500–784 −5.75%	-	-
R1	RT–198 −6.50%	198–560 −16.98%	-	-
R2	RT–213 −11.57%	213–552 −22.34%	-	-
R3	RT–214 −10.62%	214–556 −18.6%	656–773 −2.66%	-
*RfL* Dried Extract	RT–184 −5.83%	184–325 −31.85%	325–652 −61.35%	-
HG	RT–160 +1.37%	160–291 −34.79%	291–480 −53.6%	480–565 −8.46%
HG(H1)20%	RT–170 −1.04%	170–370 −26.39%	370–427 −42.22%	427–525 −15.29%
HG(H2)30%	RT–167 −1.2%	167–360 −29.44%	360–425 −40.46%	425–525 −15.02%
HG(H3)30%	RT–169 −1.77%	169–360 −48.45%	360–424 −22.99%	424–506 −11.89%
HG(R1)20%	RT–200 −3.12%	200–352 −38.75%	352–411 −22.47%	411–526 −17.24%
HG(R2)30%	RT–190 −1.24%	190–354 −41.23%	354–422 −30.4%	422–523 −15.33%
HG(R3)30%	RT–188 −2.68%	188–355 −34.1%	355–424 −32.76%	424–522 −15.03%

**Table 3:** Lattice parameters of the LDH phases in the investigated samples.

Sample	α (Å)	χ (Å)
H1	3.043	22.997
H2	3.083	22.992
H3	3.081	22.636
R1	2.960	23.084
R2	3.017	24.056
R3	3.048	23.057
HG(R1)20%	2.955	24.218
HG(R2)30%	3.013	24.971
HG(R3)30%	3.010	25.325

**Table 4:** Fitted parameters of the kinetic models used in the RfL release from composite HGs.

Sample	Zero-Order		First-Order		Higuchi		Korsmeyer-Peppas		
	k_0_, μg/mL	R^2^	k_1,_ min^−1^	R^2^	k_H_, µg/mL·min^−1/2^	R^2^	k	n	R^2^
R1	0.701	0.565	0.026	0.305	6.241	0.745	3.358	0.464	0.760
R2	0.390	0.806	0.022	0.641	3.517	0.877	3.673	0.420	0.824
R3	-	-	-	-	-	-	-	-	-
HG(R1)10%	0.338	0.760	0.020	0.6	3.059	0.83	3.654	0.387	0.787
HG(R1)20%	0.320	0.858	0.021	0.719	2.914	0.906	3.743	0.405	0.844
HG(R2)10%	0.608	0.515	0.026	0.286	5.491	0.698	3.411	0.472	0.745
HG(R2)20%	0.628	0.534	0.027	0.292	5.640	0.716	3.416	0.478	0.751
HG(R2)30%	0.714	0.602	0.028	0.325	6.281	0.775	3.401	0.484	0.779
HG(R3)10%	-	-	-	-	-	-	-	-	-
HG(R3)20%	0.273	0.813	0.019	0.67	2.501	0.867	3.717	0.368	0.808
HG(R3)30%	-	-	-	-	-	-	-	-	-

## Data Availability

The data presented in this study are available on request from the corresponding author.

## Supplementary Materials

The following supporting information can be downloaded at: https://www.mdpi.com/article/10.3390/pharmaceutics15071888/s1, Table S1. The parameters for the anthrones and derivatives (analytical reference substances) and *Frangula cortex* rich extract applied in the quantification experiments. Table S2. Phytochemical characterization of the Buckthorn bark and of the phytoextractobtainedby microwave-assisted extraction; Table S3. Chemical composition of LDH samples: metal content as determined by ICP-AES, weight loss at 450 °C for H1–H3 samples (WL_450°C_); weight gain for the samples reconstructed with *RfL* extractive solution (WG), *Rfs* content determined from UV analysis of the fresh *RfL* extractive solution and the solution recovered after the reconstruction; Figure S1 The aspect of pristine hydrogel and of composite hydrogels, Figure S2. The corresponding calibration curve obtained using standard *RfL* phytoextract solution with known concentrations; Table S4: Thermal behavior of investigated samples; Figure S3: Thermal analysis diagrams (black: TGA in %, blue: DTG in a.u. and red: DTA in volts per gram of sample) for all investigated samples categories as discussed in text: (i) as-prepared LDHs H1, H2, and H3; (ii) calcined LDHs C1, C2, and C3; (iii) LDHs reconstructed with *RfL* R1, R2, R3, and *RfL*; (iv) hydrogel HG and its composites with as-prepared LDHs HG(H1)20%, HG(H2)30%, HG(H3)30%, and (v) hydrogel composites with *RfL*-reconstructed LDHs HG(R1)20%, HG(R2)30%, and HG(R3)30%; Figure S4. SEM micrographs of lyophilized composite HGs: (a) control HG, (b–d) composites with LDHs without phytoextract, namely, (a) HG(H1)20%, (b) HG(H2)30%, (c) HG(H3)30%, and (e–g) HG composites with *RfL* phytoextract immobilized in the LDHs matrix, namely, (e) HG(R1)20%, (f) HG(R2)30%, and (g) HG(R3)30%, at different magnifications. Figure S5. The fitting of releasing experimental data by different mathematical models for sample HG(R2)20%.
