# Supplementary material for: Synergistic Sustained Drug-Release System Based on Immobilized Rhamnus frangula L. Phytoextract into Layered Double Hydroxide Covered by Biocompatible Hydrogel
Source: Pharmaceutics. 2023 Jul 5;15(7):1888. doi: 10.3390/pharmaceutics15071888 (PMC10384351; doi:10.3390/pharmaceutics15071888)
Supplement: Supplementary file 1 [file pharmaceutics-15-01888-s001.zip › pharmaceutics-2429706-supplementary.pdf]

pharmaceutics

Supplementary Material: Synergistic Sustained Drug-Release System Based on Immobilized *Rhamnus frangula* L. Phytoextract into Layered Double Hydroxide Covered by Biocompatible Hydrogel

Ana-Lorena Neaguis, Anamaria Zaharia, Octavian Dumitru Pavel, Alina Tîrşoaga, Iulia Elena Neblea, Sorin Viorel Dolana, Carmen Elena Ţebrencu, Tanta-Verona Iordache, Andrei Sârbu and Rodica Zăvoianu

1. Phytoextract preparation

Buckthorn bark (Frangulae cortex), from the trunk and branches of the shrub *Rhamnus frangula L* (*Rfl*) (Family Rhamnaceae), is considered a medicine if it has at least 2.5% anthracenosides expressed as 1,8-dihydroxymethylanthraquinone (OMA) [1]) and a content of at least 7.0% glucofrangulin, expressed as glucofrangulin A [2]. The constituents with known therapeutic activity of *RfL* bark are emodin-di- and mono-glycosides i.e. the diglycosides glucofrangulin A (emodin-6-0-α-L-rhamnosyl-8-0-β-D-glucoside) and glucofrangulin B (emodin -6-0-β-D-apiosyl-8-0-β-D-glucoside) and frangulin monoglycosides A, B, C (emodin-6-0-α-L-rhamnoside, emodin-6-0-β- D-apioside, emodin-6-0-β-D-xyloside) and emodin-8-0-β-D-glucoside. The *RfL* bark influences the excretion functions of the human body (laxation, diuresis) and it is administered as a laxative or purgative, depending on the dose [3].

Vegetal material: Buckthorn bark used was collected in April 2020 (manually), from the Bistrita area, Bistrita-Nasaud county (from spontaneous flora) by SC HERBARIUM VERONICAE SRL, Mures county. The botanical identification was carried out according to the Manufacturer's Technical Data Sheet and "Cx. *Frangula cortex*", *Romanian Pharmacopeioa* [1] for naturally dried plant material. The evaluation of the buckthorn bark took into account the quality requirements of plant material for the extraction of anthraquinone compounds for medical use; the purity conditions were verified according to the provisions of the *Romanian Pharmacopoeia*, ch. IX D.4, ch. IXD.4 and *European Pharamacopoeia* 6.0, chap. 2.8.2 [1,2] The evaluation of the level of microbial contamination was made based on the criteria/specifications of the *European Pharmacopoeia* - edition 6.0 [2] and specific standards in force regarding microbial contamination. The dried material was grinded in a fine powder and the samples were stored in airtight bottles, in a dark place at 4°C.

Chemicals: All the chemicals were purchased from Sigma Aldrich and Chimreactiv, used as received, without further purification or distillation. The solutions were prepared using distilled water.

Extraction procedure: The extracts from the buckthorn bark were obtained by microwave-assisted extraction/ MAE (230V-50Hz), with the ecological solvent ethanol 70%v/v obtained from ethyl alcohol refined from cereals min. 96% v/v and purified water, according to the *Romanian Pharmacopoeia*, X th edition. The extraction parameters were:

1: 8 solid to liquid ratio, wt/v

5 minutes extraction time

400 W microwave iradiation power.

The extraction mixture was filtered through textile filter yielding the enriched phytoextract in oxymethylanthraquinones. The crude extract was stored at 4°C before performing different bioassays and phytochemical analysis.

Sample analysis*:* The high-performance thin-layer chromatography (HPTLC) identification of anthrones and derivatives from buckthorn bark and phytoextract was performed using adapted validated HPTLC method [3,4]. In brief, first, it was fingerprinted using HPTLC and the resulting data (*Rf* values and colour hues) were

verified against the following standards: Glucofrangulin A (emodin-6-0-α-L-ramnosil-8-0-β-D-glucosyde), Frangulin A (emodin-6-0-α-L-ramnozide), Aloin (Barbaloin), Emodin (1,6,8-trihydroxy-3-methyl-anthraquinone). Potential matches were confirmed by spectral overlay analysis.

The CAMAG HPTLC system (Switzerland) used in this study consisted of a CAMAG TLC Reprostar 3 visualizer with 12 bit digital camera and 16 mm lens CAMAG, Linomat IV semi-automatic sample applicator and ADC2 automated development chamber, a CAMAG TLC scanner 3. The system was operated by WinCATS Version 1.4.3 software, which controls all chromatographic operations and analyses. In order to perform the identification of anthrones and derivatives, the sample was subjected to the same HPTLC conditions, using the solvent system: ethyl acetate: methanol: water in a proportion of 20: 2.7: 2 (v/v/v), as well as 8.0% potassium hydroxide solution in ethanol as derivatizing reagent. The HPTLC image obtained after derivatization with identification reagents indicates the separation of reference substances and constituents from the analyzed samples. The *Rf* values for the reference substances were identified by examination at 366 nm, after derivatization. Following the visualization at 366 nm, after sprinkling with the identification reagent, at distinct *Rf* values and by referring to the reference substances, the specific constituents of the plant material and the two plant extracts were identified (table S1).

Table S1. The parameters for the anthrones and derivatives (analytical reference substances) and *Frangula cortex* rich extract applied in the quantification experiments.

Sample/Analytical reference substance	Purity (%)	Supplier	The applied volume (μL)	Concentration (mg/mL)
Buckthorn bark ethanolic extract	-		10	-
Buckthorn bark methanolic extract	-		10	-
Buckthorn bark MAE extract	-		10	-
Glucofrangulin A, CAS No. 21133-53-9 Product No. 84189	>90	PhytoLab GmbH & Co KG, Germany	10	1
Frangulin A, CAS No. 521-62-0 Product No. 0435	≥95	Extrasynthese Genay, France	10	1
Aloin, CAS No.1415-73-2 Product No. 06088	Analytical standard	Sigma-Aldrich, St. Louis,Switzerland	10	1
Emodin, CAS No. 518-82-1 Product No. E7881	>90	Sigma-Aldrich, St. Louis, Missouri, USA	10	1

The identification chromatogram of anthrones and derivatives is relevant after derivatization at λ=366 nm. So, at *Rf* = 0.25 distinct orange-brick colored bands are identified, corresponding to the glucofrangulin A standard; at *Rf* = 0.72 distinctly pronounced bands of orange-brick color can be identified, corresponding to the frangulin A standard; at *Rf*= 0.90-0.91, pronounced distinct bands of intense brick color corresponding to the emodin standard can be identified; at *Rf*=0.31and 0.78 (visible, after derivatization) distinct bands of brick color can be identified, without a correspondent in the applied standards, from the category of anthrones.

The qualitative analyses have been performed according to the methods described in the literature [5,6,7] and 10th Edition of Romanian Pharmacopoeia , Chapter IX. C.15, C.17, D.8.[1] The concentration of oxymethylanthraquinones (OMA) was expressed as 1,8-dihydroxymethylanthraquinones. The characteristics of the raw material and phytoextract are presented in Table S2.

Table S2. Phytochemical characterization of the Buckthorn bark and of the phytoextract obtained by microwave assisted extraction.

Characteristics	Vegetable raw material - buckthorn bark	Phytoextract from Buckthorn bark	Analytical Method
Organoleptic characteristics	External surface grey-brown, whitish lenses, odorless, bitter taste. Degree of comminution of vegetable product, mm=0.4-0.6	Color dark brown, clear, characteristic smell, slightly astringent taste	cf. Romanian Pharmacopoeia - 10th edition [1]
pH	-	6.17	potentiometric method
Density, g/mL	-	0.8985	densimeter
Evaporation residue, wt %	-	2.25	cf. Romanian Pharmacopoeia - 10th edition [1]
Humidity, wt. %	9.94	-	cf. Romanian Pharmacopoeia - 10th edition [1]
Total ash, wt. %	4.42	-	cf. Romanian Pharmacopoeia - 10th edition [1]
Extractible in water wt. % *from vegetal product*	19.58		cf. Romanian Pharmacopoeia - 10 th edition [1]
Extractible in ethanol 70% v/v wt. % *from vegetal product*	21.31		cf. Romanian Pharmacopoeia - 10th edition [1]
Extracted substances in ethanol 70% v/v wt. % *from dried substance*		22.27	cf. Romanian Pharmacopoeia - 10th edition [1]
oxymethylanthraquinones (OMA), expressed as *8-dihydroxymethylanthraquinones.*	3.572 *wt* % *calculated to the vegetable product.*	0.256 *wt* % *calculated to the solution* 11.40 *wt* % *calculated to the dried substance*	cf. Romanian Pharmacopoeia - 10th edition [1]
Total polyphenols, expressed as *gallic acid*	6.79 *wt* % *calculated to the vegetable product*	0.311 *mg/ mL of solution* 13.84 *wt* % *calculated to the dried substance*	Modified method Singleton-Rossi method with Folin-Ciocalteu reagent [5,6]
Total Tannins, expressed as *tannic acid*	0,6045 *wt* % *calculated to the vegetable product.*	0.0005 *mg/ mL* 2.339 wt. % *calculated to the dried substance*	Adapted method -Reaction with Folin reagent and Malvezin reagent [7]

Buckthorn bark ethanolic extract is well known and much used as dietary supplements [8, 9, 10], so that no *in vitro* or *in vivo* tests were necessary.

2. Chemical composition of LDH samples

Table S3. Chemical composition of LDH samples: metal content as determined by ICP-AES, weight loss at 450°C for H1-H3 samples ($WL_{450°C}$); weight gain for the samples reconstructed with *RfL* extractive solution (WG), *RfL* content determined from UV analysis of the fresh *RfL* extractive solution and the solution recovered after the reconstruction .

Sample	Metal content (wt. %)				$WL_{450°C}$	*RfL* content	Molar ratios		
	Mg	Ca	Fe	Al	(wt. %)	(wt. %)	(Mg+Ca)/(Fe+Al)	Mg/Ca	Fe/Al
H1	18.2	-	21.1	-	35.4	-	1.99	-	-
H2	18.2	-	16.8	2.0	36.8	-	2.00	-	4.05
H3	16.1	2.9	16.6	2.0	33.9	-	1.98	9.14	4.00
C1	28.2		32.5		-	-	2.00	-	-
C2	28.8		26.6	3.2	-	-	1.99	-	4.00
C3	24.5	4.4	25.1	3.0	-	-	1.99	9.16	4.03
R1	19.8		22.8		29.8	10.9	2.00	-	-
R2	16.6		15.6	1.8	42.2	10.3	1.98	-	4.06
R3	15.1	2.7	15.5	1.8	38.4	10.2	2.00	9.16	4.03

$Mg(NO_3)_2x6H_2O$, $Ca(NO_3)_2x4H_2O$, $Fe(NO_3)_3x9H_2O$, $Al(NO_3)_3x9H_2O$, NaOH and Na_2CO_3 p.a. grade from Merck (Darmstadt, Germany) and distilled water were used for the preparation of LDH type compounds.

3. Preparation of Composite Hydrogels

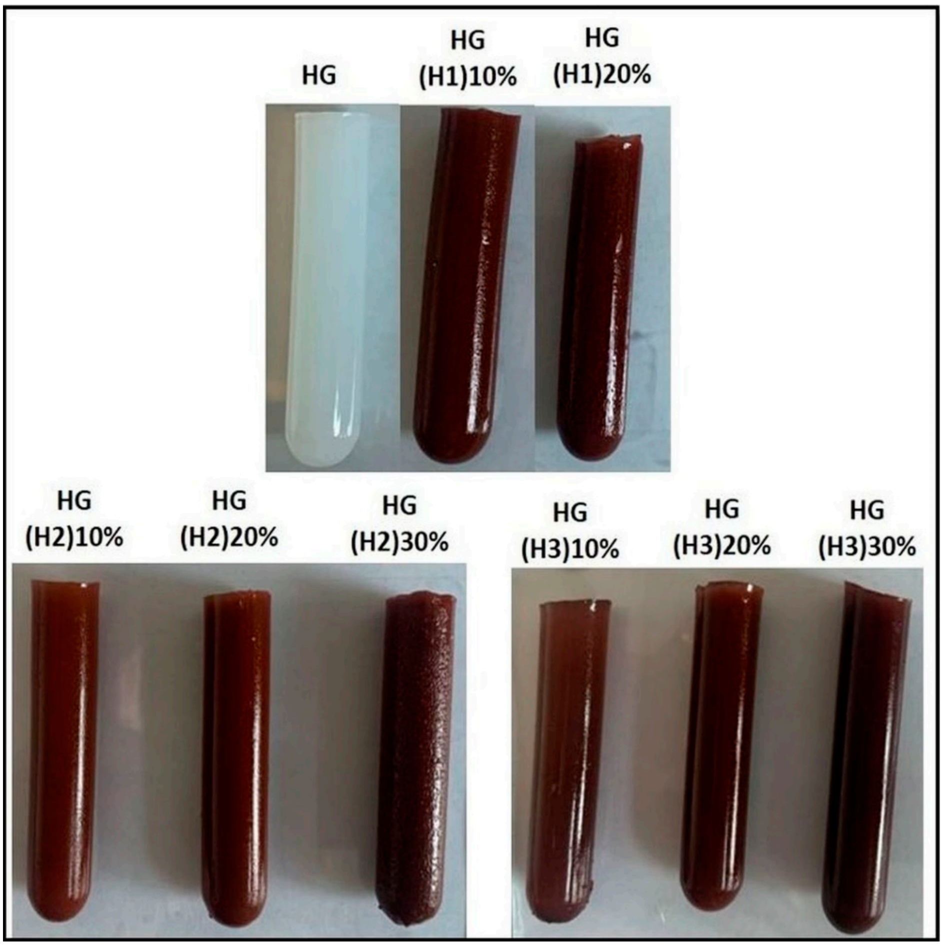

Figure S1. The aspect of pristine hydrogel and of composite hydrogels.

4. Determination of *Rhamnus frangula L* (*RfL*) phytoextract released in vitro from LDH*RfL* composites and HG LDH*RfL* composites

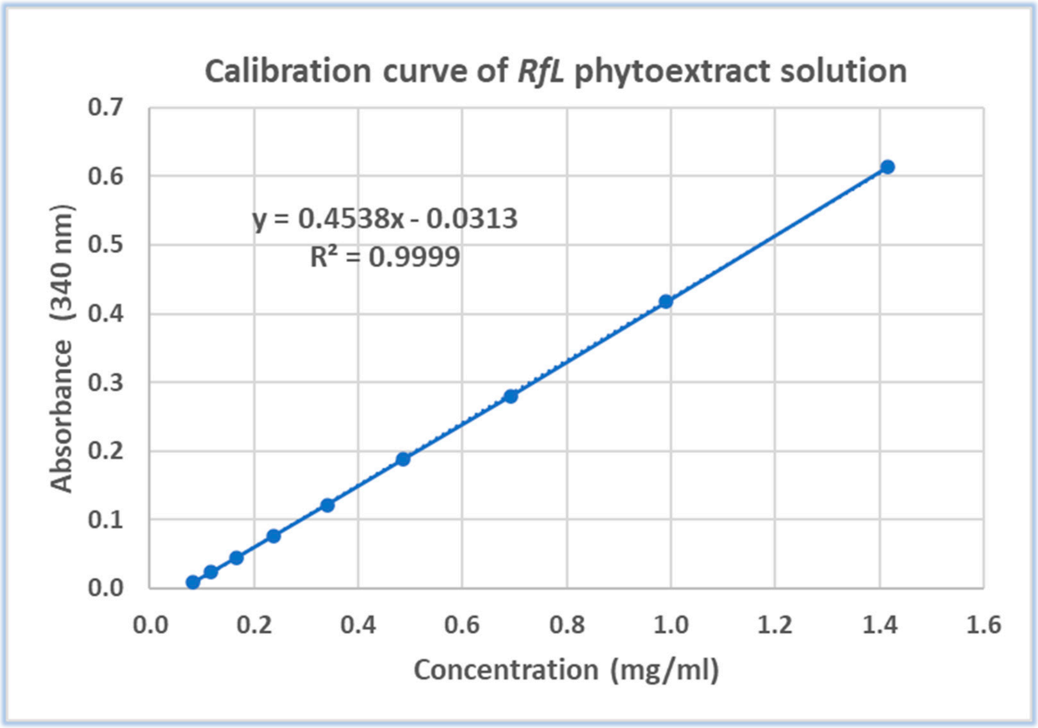

Figure S2. The calibration curve obtained using standard *RfL* phytoextract solution with known concentrations.

5. Thermal analysis

Following the logic of the synthesis protocol, five categories of samples were investigated by thermal analysis: i) as-prepared LDH (H1-H3), ii) air-calcined (at 450^{0}C) samples that served as precursors for the reconstruction process (C1-C3), iii) LDHs reconstructed with *RfL* (R1-R3), iv) composite samples featuring hydrogel with as-prepared LDH (HG(H1)20%; HG(H2)30%; HG(H3)30%), and v) composite samples of hydrogel with *RfL* reconstructed LDH (HG(R1)20%; HG(R2)30%; HG(R3)30%). Weight losses observed in the TGA curves for all investigated samples are shown in Table S4, as values extracted from the thermal analysis diagrams given in Figures S2.

As a general thermal behavior (Table S4), LDH and LDH*RfL* (samples H1-H3; R1-R3) share a common feature consisting of three main decomposition steps whereas only two steps are observed for the calcined LDH samples and an additional fourth step only for hydrogel-containing composites.All as-prepared LDH samples, namely: H1 (Mg_2Fe), H2 ($Mg_2Fe_{0.8}Al_{0.2}$) and H3 ($Mg_{1.8}Ca_{0.2}Fe_{0.8}Al_{0.2}$) share a first overall weight loss step (endothermic) up to about 220^{0}C where the elimination of water occurs: first as the evaporation of powder-trapped moisture, followed by the removal of chemically bound water present in the LDH structure as water (dehydration) and subsequent start of the hydroxyl moieties removal (dehydroxylation) **[11]**. The substitutions with Al and Ca performed in the LDH structure are correlated with a larger water loss in this first step, in line with the higher content of Al/Ca substituent cations with different temperature-dependent affinity for water than the original cations. The second endothermic decomposition step continues the removal of hydroxyl moieties from the LDH, together with the subsequent release of some CO_2 in its incipient carbonate decomposition phase, process that is still unable to determine the collapse of the LDH layered structure, up to around 500^{0}C **[11]**. The substitution with Al does not induce in this step a significant weight loss difference compared to the unsubstituted composition, whereas the smaller

weight loss observed for the Ca-containing sample could be correlated with the larger number of weaker-bound species of water already removed in the previous decomposition step at lower temperatures. The last endothermic weight loss step, above 500⁰C, consists of entire carbonate content decomposition to a mixture of oxides, process that destroys the layered structure without any chance of further chemical reconstruction of the original LDH.

Table S4. Thermal behavior of investigated samples.

Sample	$T_i - T_f$ (⁰C) Δm (%)	Thermal effect/ T_{max}	$T_i - T_f$ (⁰C) Δm (%)	Thermal effect/ T_{max}	$T_i - T_f$ (⁰C) Δm (%)	Thermal effect/ T_{max}	$T_i - T_f$ (⁰C) Δm (%)	Thermal effect/ T_{max}
H1	RT-209 -11.41 %	Endo/200	209-500 -22.42 %	Endo/290; 375	500-569 -5.01 %	Endo/515; 564		
H2	RT-223 -13.20 %	Endo/212	223-494 -22.71 %	Endo/293; 398	494-543 -2.79 %	Endo/502		
H3	RT-231 -14.00 %	Endo/224	223-550 -19.74 %	Endo/395	664-773 -2.92 %	Endo/745		
C1	RT-500 -2.4 %		500-784 -1.25 %	Endo/728				
C2	RT-500 -3.2 %		500-784 -1.65 %	Endo/717				
C3	RT-500 -2.5 %		500-784 -5.75 %	Endo/602; 763				
R1	RT-198 -6.50 %	Endo <100	198-560 -16.98 %	Exo/335; 425; 465				
R2	RT-213 -11.57 %	Endo <100	213-552 -22.34 %	Exo/348; 433				
R3	RT-214 -10.62 %	Endo <100	214-556 -18.6 %	Exo/345; 440	656-773 -2.66 %	Endo/750		
Rfs (*RfL* dried extract)	RT-184 -5.83 %	Endo <100	184-325 -31.85 %	Exo/229	325-652 -61.35 %	Exo/565		
HG	RT-160 +1.37 %	Endo <100	160-291 -34.79 %	Exo/210	291-480 -53.6 %	Exo/356; 431	480-565 -8.46 %	Exo/517
HG(H1)20%	RT-170 -1.04 %	Endo <100	170-370 -26.39 %	Exo/182	370-427 -42.22 %	Exo/405	427-525 -15.29%	Exo/473
HG(H2)30%	RT-167 -1.2 %	Endo <100	167-360 -29.44 %	Exo/186	360-425 -40.46 %	Exo/405	425-525 -15.02%	Exo/473
HG(H3)30%	RT-169 -1.77 %	Endo <100	169-360 -48.45 %	Exo/190	360-424 -22.99 %	Exo/405	424-506 -11.89 %	Exo/473
HG(R1)20%	RT-200 -3.12 %	Endo <100	200-352 -38.75 %	Exo/215; 292	352-411 -22.47 %	Exo/407	411-526 -17.24 %	Exo/477
HG(R2)30%	RT-190 -1.24 %	Endo <100	190-354 -41.23 %	Exo/214	354-422 -30.4 %	Exo/409	422-523 -15.33 %	Exo/477
HG(R3)30%	RT-188 -2.68 %	Endo <100	188-355 -34.1 %	Exo/206	355-424 -32.76 %	Exo/407	424-522 -15.03 %	Exo/477

Each substitution of Fe with Al determines a decrease of -56 + 27 = -29 atomic mass units (amu) for the cations composition of the LDH unit, hence the decomposition of a similar mole fraction of carbonate from the reference Mg_2Fe hydrotalcite should be characterized by a smaller weight loss than the substituted $Mg_2Fe_{0.8}Al_{0.2}$ composition. The experimental data exhibit a significant opposite behavior, the unsubstituted Mg_2Fe composition loses almost double the amount of CO_2 than the substituted $Mg_2Fe_{0.8}Al_{0.2}$ LDH. Such a behavior may indicate that the carbonate content playing the role of building entities of the LDH structure decreases with the substitution with Al, probably due to an increased water affinity (as observed in the first decomposition step) and/or a possible coexistence of Fe^{II} together with the majority Fe^{III} species so that a decrease of the overall cationic charge would accommodate a lower amount of carbonate anions. An indication suggesting the above phenomena is the temperature range where the last carbonate decomposition step occurs: 500-569^{0}C for Mg_2Fe reference and a lower (thus less stable) 494-543^{0}C for $Mg_2Fe_{0.8}Al_{0.2}$ sample (Table S4). A further substitution of Mg with Ca determines an increase of -24 + 40 = +16 amu for the cations composition of the LDH unit compared to the Mg_2Fe reference, and, as reasoned before, a smaller CO_2 weight loss in this last decomposition step is expected, in line with the experimental observations. It is noteworthy that in this last step CO_2 weight loss is comparable for $Mg_2Fe_{0.8}Al_{0.2}$ and $Mg_{1.8}Ca_{0.2}Fe_{0.8}Al_{0.2}$, showing that the substitution of Mg^{II} with Ca^{II} induces no changes in the overall charges balance and the carbonate anions content should be similar. However, the main difference generated by the Ca substitution is the shift towards significantly higher temperatures of the last carbonate decomposition step (664-773^{0}C *vs.* 494-543^{0}C) showing thus an important stabilization effect of Ca upon the layered LDH structure.

For the calcined LDH samples C1-C3 the overall weight losses are small, as expected for the above described samples which were submitted to a thermal treatment at 450^{0}C under air. All calcined samples were kept in a vacuum desiccator but still, minute amounts of water (released under 500^{0}C) and CO_2 were trapped during the samples manipulation in the ambient laboratory atmosphere. As expected, keeping the samples in the vacuum desiccator does not entirely prevent a slow CO_2 uptake (originating from repeated openings of the desiccator to access other samples) which is the greatest for Ca-containing LDH, as a consequence of its higher affinity towards CO_2 that strengthen the layered structure.

The LDH samples reconstructed by immersion of calcined samples in *RfL* extractive solution (R1-R3) present the same first endothermic water removal step (up to about 210^{0}C) as in the case of as-prepared LDH samples (H1-H3). The main systematic difference compared to the reference as-prepared LDHs is the smaller weight loss, indicating that molecules with lower volatility than water were incorporated into the samples. Indeed, the second decomposition step features two exothermal peaks located at around 335 and 435^{0}C that can only originate from the oxidative degradation of an organic component. As in the case of the first decomposition step, the comparisons with the as-prepared LDHs show noticeable smaller weight losses for all reconstructed samples, phenomenon suggesting that organic molecules (significantly larger than water) were incorporated and limited the availability of the LDH structure to re-accommodate the initial amount of (small) water molecules present in the reference as-prepared LDHs. The weight loss of the reference as-prepared Mg_2Fe composition is 27.45% (second and third steps) compared to 16.98% for the *RfL* reconstructed Mg_2Fe sample (R1), hence a difference of -10.45%. The same calculation performed for $Mg_2Fe_{0.8}Al_{0.2}$ and $Mg_{1.8}Ca_{0.2}Fe_{0.8}Al_{0.2}$ leads to differences of -3.16% and -1.14%, respectively. Although these differences cannot be attributed entirely to the organic fraction (water molecules would also be included), the amplitude of the exothermal DTA peak varies in the same order, suggesting that the largest amount of *RfL* incorporated during the reconstruction is found in the Mg_2Fe composition, followed by $Mg_2Fe_{0.8}Al_{0.2}$ and $Mg_{1.8}Ca_{0.2}Fe_{0.8}Al_{0.2}$. Such a variation may be correlated with the rigidity or the stability of the layered hydrotalcite structure, where the cationic substitutions (especially with Ca, that still presents the last high-temperature carbonate

decomposition step) allow a lower amount of large organic molecules incorporated between the hydrotalcite layers. As a reference, a sample of *RfL* dried extract (*Rfs*) has been included along with the LDH*RfL* samples. The *Rfs* sample follows a decomposition pattern with three steps: a first endothermic (water removal), followed by two (exothermal) oxidative degradation steps with an absolute DTA maximum at 566^{0}C. The incorporation of the organic content from *RfL* extractive solution in the inorganic LDH framework significantly decreases the temperature of the exothermal degradation steps to 335 and 435^{0}C, phenomenon that is generally common in the thermal analysis of organic-inorganic composites due to the uniform spread of the organic component in the inorganic matrix and also due to a potential catalytic effect of the transition metal(s) content in the high-temperature air-based oxidation processes.

The analysis of the thermal behavior of the reference PEG-DA hydrogel shows that this sample exhibits a unique feature of weight gain (+1.37 %) up to around 160^{0}C. Reports of DSC studies performed in air atmosphere on PEG [12] show that the degradation of the polyether backbone can be initiated at low temperatures, under 180^{0}C. Degradation of the same polyether backbone in PEG-DA samples investigated by Browning *et al.* [13] concludes the oxidative nature of this degradation process. The conclusions of these literature reports may suggest that our PEG-DA hydrogel sample suffers an oxidative degradation process accompanied with oxygen incorporation, as sustained by the strong exothermal evolution of the DTA signal. This first degradation step is followed by three exothermal steps with weight losses, accounting for further degradation of the polymeric matrix [14] that follows an oxidative path due to the static air atmosphere around the crucible that holds the sample. The other three samples in this category, composite hydrogel with as-prepared LDHs, exhibit for the first degradation step (under 170^{0}C) exothermal effects (as in the case of the reference hydrogel), but only weight losses. The mass fraction of the hydrogel in these composites is 70 or 80%, hence the weight gain of +1.37% of the hydrogel alone can be compensated by the weight loss of each as-prepared LDH component, calculation giving -1.19% (HG(H1)), -3.0% (HG(H2)) and -3.2% (HG(H3)). All these calculated values are higher than the corresponding ones reported in the second column of Table S4 and therefore cannot exclude nor confirm the coexistence of both processes of: oxidative degradation of the PEG-DA hydrogel and respectively the dehydration of the as-prepared LDH. However, the fact that the experimental weight losses are smaller than the above-calculated values suggests that the interaction between the hydrogel and the LDH(s) stabilizes in some extent the composite system. The second and third exothermal decomposition steps feature a smaller weight loss for the second step than for the third step for the composites containing Mg_2Fe and $Mg_2Fe_{0.8}Al_{0.2}$ LDHs, in line with the values observed for the reference hydrogel. The notable exception is $Mg_{1.8}Ca_{0.2}Fe_{0.8}Al_{0.2}$-based composite which has an opposite behavior, losing more weight in the second step than the third, while keeping the same exothermal nature of the processes. Such an observation suggests that the presence of Ca^{II} induces a different interaction of the LDH with the polymeric matrix. The last exothermal decomposition step occurs in a similar way for all three composite samples but the main difference between the composites and the reference hydrogel is that the maximum of the DTA exothermal peak is located at lower temperature for the composites, which suggests that the oxygen required for the last burning step of the carbonaceous residue originating from the polymeric matrix can be extracted from the highly reactive metal oxide species generated immediately after CO_2 elimination from carbonate: it has been reported that bulk Fe_2O_3 can be reduced by carbonaceous residues from the polymeric matrix at temperatures as low as 470^{0}C [15], most probably to Fe_3O_4 in a first stage (under 570^{0}C) [16] that may subsequently evolve towards other compositions of the Fe-O phase diagram [15].

The hydrogel encapsulated *RfL* reconstructed LDHs (e.g. HG(R1-R3)), show a similar thermal behavior (four decomposition steps) with the HG(H1-H3) samples. The first exothermal decomposition step presents slightly larger weight losses than HG(H1-H3). In contrast, for the second and third exothermal decomposition steps an opposite behavior

is observed: the presence of the organic components from *RfL* induces a larger weight loss in the second step than in the third degradation step of the composite systems. Another difference when compared to the composites without *RfL* is the $Mg_{1.8}Ca_{0.2}Fe_{0.8}Al_{0.2}$-based system: the effect of Ca that determined a distinctive behavior in the previous category (higher weight loss in the second step than in the third) is almost canceled; the organic components from the extractive solution of *RfL* determine now a more uniform thermal behavior in the same category of samples, despite the presence of only a small over-weight loss in the second *vs.* third decomposition step.

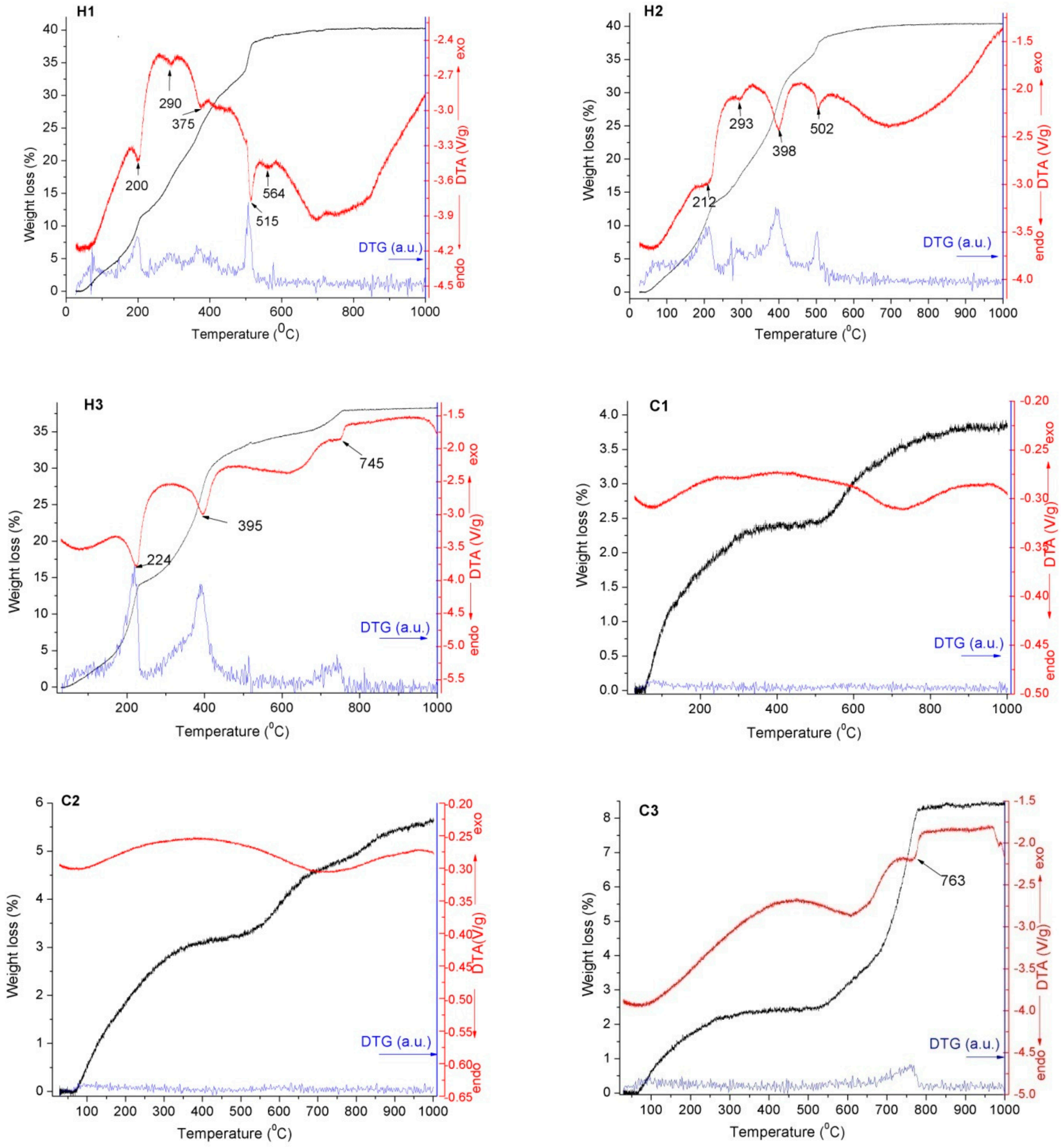

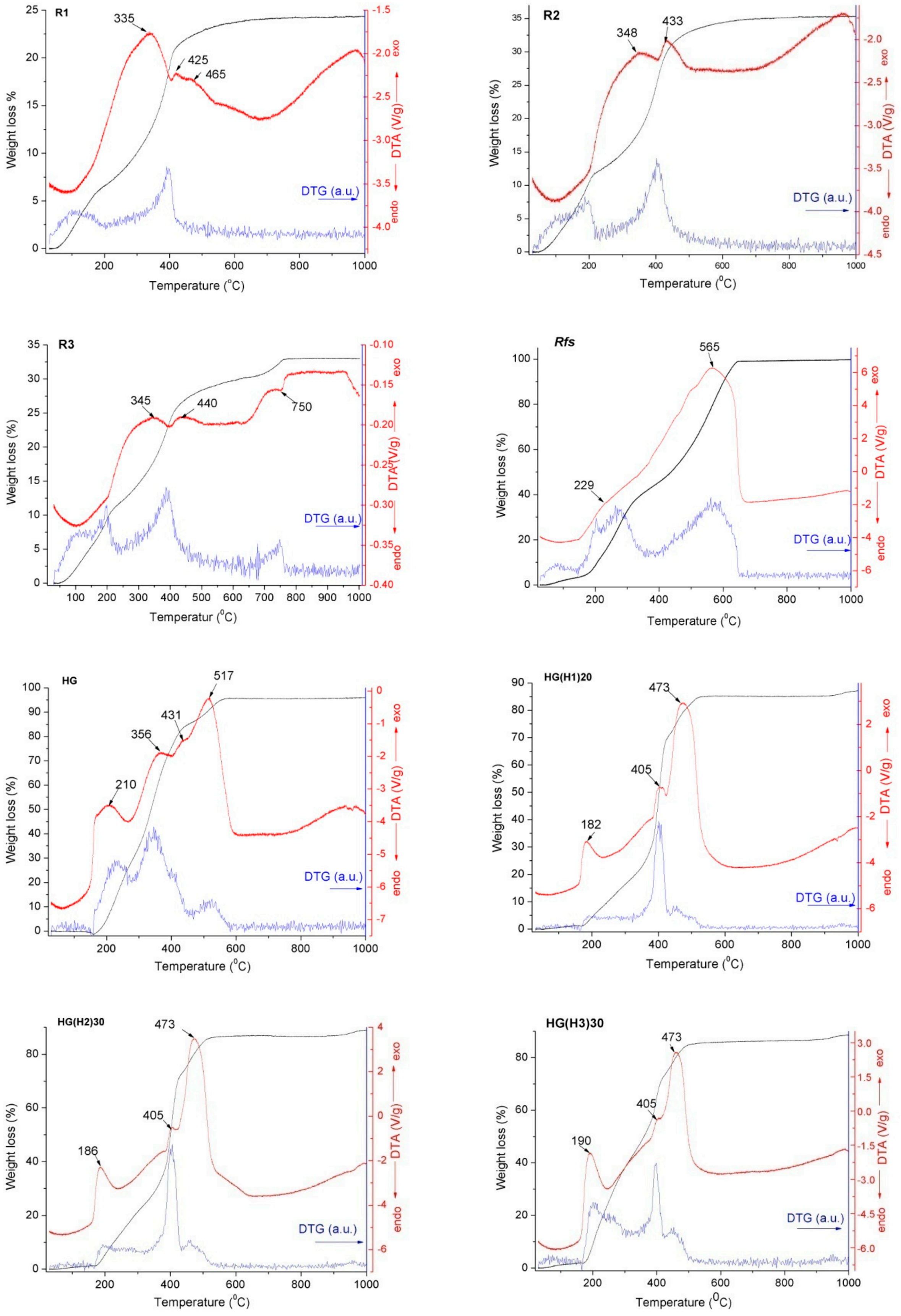
R1
335
425
465
Weight loss %
Temperature (°C)
DTA (V/g)
exo
endo
DTG (a.u.)
R2
348
433
Weight loss (%)
Temperature (°C)
DTA (V/g)
exo
endo
DTG (a.u.)
R3
345
440
750
Weight loss (%)
Temperatur (°C)
DTA (V/g)
exo
endo
DTG (a.u.)
Rfs
565
229
Weight loss (%)
Temperature (°C)
DTA (V/g)
exo
endo
DTG (a.u.)
HG
517
431
356
210
Weight loss (%)
Temperature (°C)
DTA (V/g)
exo
endo
DTG (a.u.)
HG(H1)20
473
405
182
Weight loss (%)
Temperature (°C)
DTA (V/g)
exo
endo
DTG (a.u.)
HG(H2)30
473
405
186
Weight loss (%)
Temperature (°C)
DTA (V/g)
exo
endo
DTG (a.u.)
HG(H3)30
473
405
190
Weight loss (%)
Temperature (°C)
DTA (V/g)
exo
endo
DTG (a.u.)

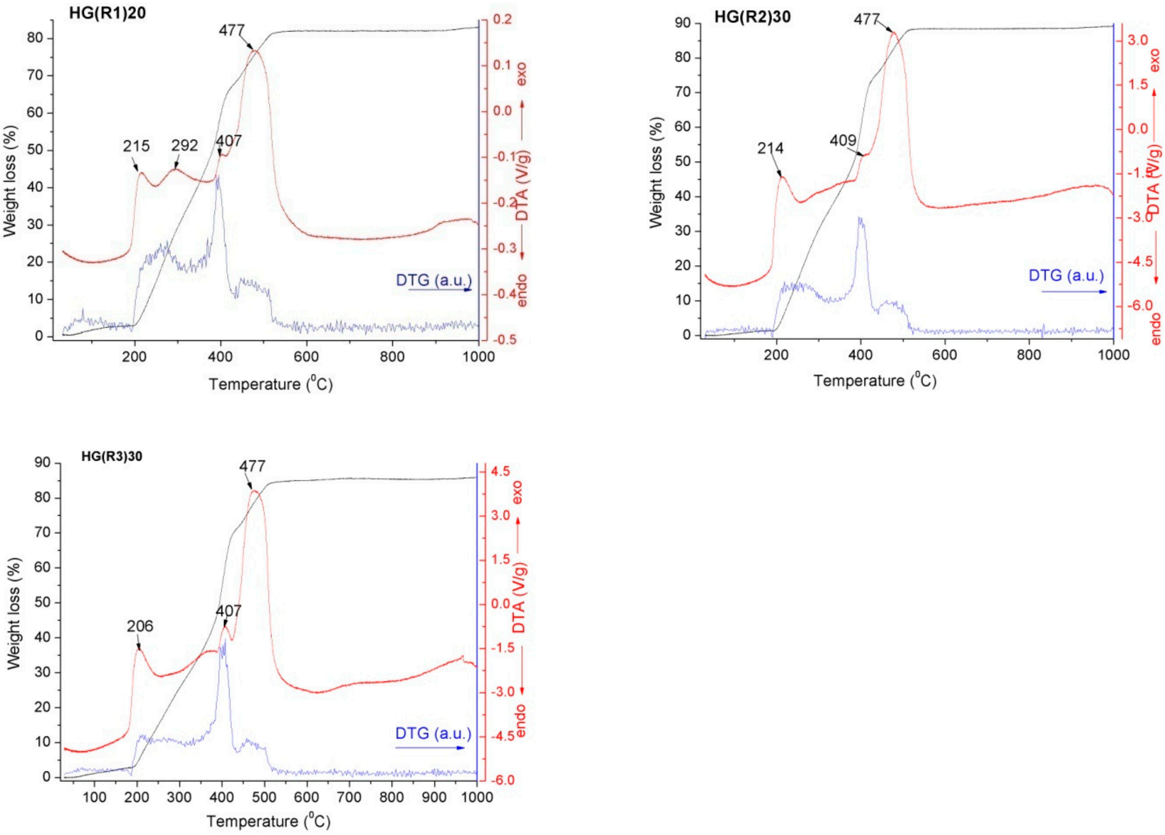

Figure S3. Thermal analysis diagrams (black: TGA in %, blue: DTG in a.u. and red: DTA in volts per gram of sample) for all investigated samples categories as discussed in text: i) as-prepared LDHs H1, H2, H3; ii) calcined LDHs C1, C2, C3; iii) LDHs reconstructed with *Rfs* R1, R2, R3 and *Rfs*; iv) hydrogel HG and its composites with as-prepared LDHs HG(H1)20%, HG(H2)30%, HG(H3)30% and v) hydrogel composites with *Rfs*- reconstructed LDHs HG(R1)20%, HG(R2)30%, HG(R3)30%.

6. SEM analyses

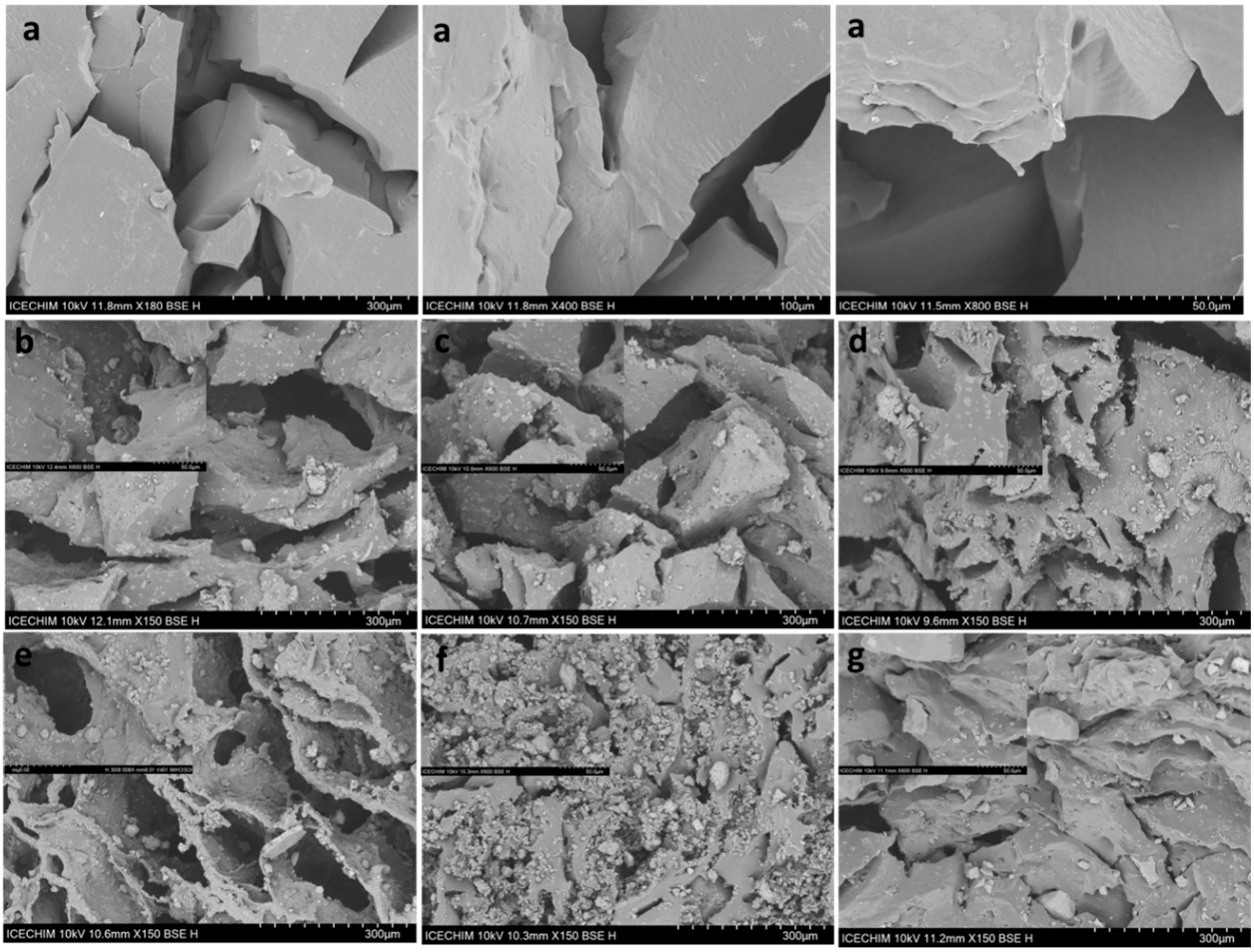

Figure S4. SEM micrographs of lyophilized composite HGs: (a) control HG, (b) - (d) composites with LDHs without phytoextract, namely: (a) HG(H1)20%, (b) HG(H2)30%, (c) HG(H3)30% and (e) - (g) HG composites with RfL phytoextract immobilized in the LDHs matrix namely :(e) HG(R1)20%, (f) HG(R2)30%, (g) HG(R3)30%, at different magnifications.

7. Releasing Models

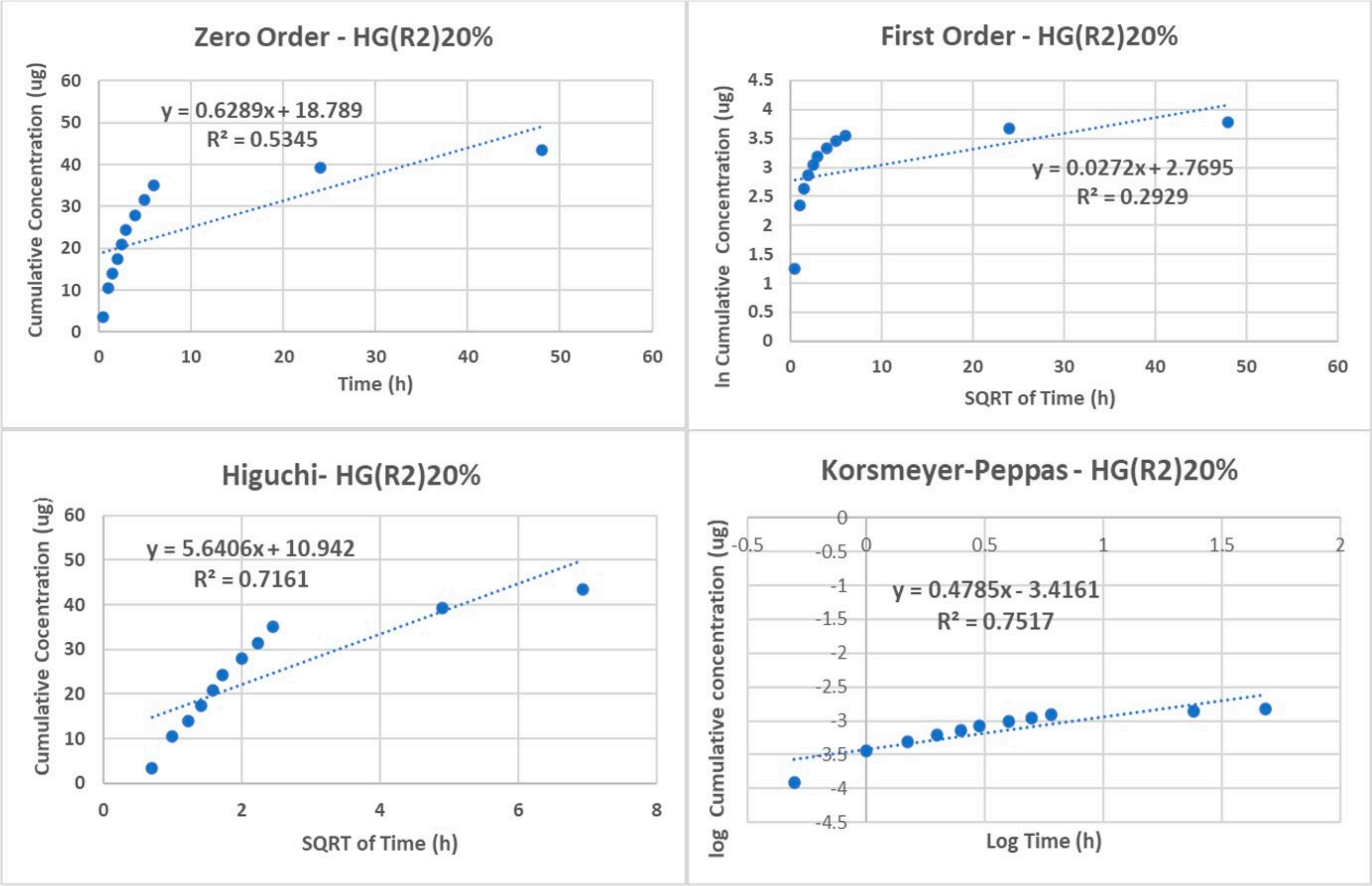

Figure S5. The fitting of releasing experimental data by different mathematical models for sample HG(R2)20%.

References

1. *Romanian Pharmacopoeia*, 10th ed., Chapter VIII "*Frangula bark*" , Editura Medicala, Bucuresti , 2000: 427
2. *European Pharmacopoei*a, 6th ed., Chapter 6.8 "*Frangulae cortex*" Council of Europe, Strasbourg, France, 2008: 1516
3. Wagner, H.; Bladt, S. Plant Drug Analysis: *A Thin Layer Chromatography Atlas*, 2nd ed.; Springer: Dordrecht , Netherland; New York, USA, 2009.
4. Reich, E.; Schibli, A. *High-Performance Thin-Layer Chromatography for the Analysis of Medicinal Plants*; 1th ed. Thieme: New York, USA, 2007.
5. Ainsworth, E.A.;Gillespie, K.M., Estimation of total phenolic content and other oxidation substrates in plant tissues using Folin-Ciocalteu reagent, *Nature Protocols*, **2007**, 2, 875-877, doi: 10.1038/nprot.2007.102
6. Everette, J.D.;Bryant, Q.M.;Green, A.M.; Abbey, Y.A.;Wanglia, G.W.; Walker, R.B., Thorough study of reactivity of various classes toward the Folin-Ciocalteu reagent. *J. Agr. Food Chem*. **2010**, 58, 8139-81, doi: 10.1021/jf1005935
7. Wiesneth, S,; Jürgenliemk,G., *Total phenolic and tannins determination: a modification of Ph. Eur. 2.8.14 for higher throughput*, *Pharmazie* **2017**, 72, 195–196,doi: 10.1691/ph.2017.6911
8. EMA (European Medicines Agency), *Assessment report on Rhamnus frangula L. cortex,* EMA/HMPC/483550/ 2018, 2019, Committee on Herbal Medicinal Products (HMPC), www.ema.europa.eu/contact
9. Paneitz, A., Westendorf, J. , Anthranoid contents of rhubarb (Rheum undulatum L.) and other Rheum species and their toxicological relevance, *Eur. Food Res. Techn*., **1999**, 210: 97–101, doi: 10.1007/s002170050542
10. Rauwald H.D., *Phytomedicines of Europe, Chapter 9: Herbal Laxatives: Influence of Anthrones—Anthraquinones on Energy Metabolism and Ion Transport in a Model System*, Am. Chem. Soc., New York, USA, 1998: 97-116, doi: 10.1021/bk-1998-0691.ch009
11. Hajek M., Kocik J., Frolich K., Vavra A., "Mg-Fe mixed oxides and their rehydrated mixed oxides as catalysts for transesterification", *J. Clean. Prod*., **2017**, *161*, 1423-1431, DOI: 10.1016/j.jclepro.2017.05.199.
12. Royer A., Barriere T., Gelin J.C., "The degradation of poly(ethylene glycol) in an Inconel 718 feedstock in the metal injection moulding process", *Powder Techn*., **2015**, *284*, 467-474, doi: 10.1016/j.powtec.2015.07.032.

13. Browning M.B., Cereceres S.N., Luong P.T., Cosgriff-Hernandez E.M., "Determination of the in vivo degradation mechanism of PEGDA hydrogels.", *J. Biomed. Mater. Res. A.*, **2014**, *102*, 4244-4251. doi: 10.1002/jbm.a.35096.
14. Neblea I.E., Gavrila A.-M., Iordache T.-V., Zaharia A., Stanescu P.O., Radu I.-C., Burlacu S. G., Neagu G., Chiriac A.-L., Sarbu A., "Interpenetrating networks of bacterial cellulose and poly (ethylene glycol) diacrylate as potential cephalexin carriers in wound therapy.",*J. Polym. Res.*, **2022**, *29*, 406, 1-12. DOI: 10.1007/s10965-022-03250-9
15. Murakami T., Kasai E., "Reduction Mechanism of Iron Oxide–Carbon Composite with Polyethylene at Lower Temperature",*ISIJ International*, **2011**, *51*, 9-13, DOI: 10.2355/isijinternational.51.9
16. Spreitzer, D. and Schenk, J. "Reduction of Iron Oxides with Hydrogen - A Review." *Steel Res. Int.*, **2019**, 90: 1900108. DOI: 10.1002/srin.201900108
